# Combined Analysis of Volatile Terpenoid Metabolism and Transcriptome Reveals Transcription Factors Related to Terpene Synthase in Two Cultivars of *Dendrobium officinale* Flowers

**DOI:** 10.3389/fgene.2021.661296

**Published:** 2021-04-22

**Authors:** Ninghong Li, Yingxue Dong, Min Lv, Li Qian, Xu Sun, Lin Liu, Yongping Cai, Honghong Fan

**Affiliations:** School of Life Sciences, Anhui Agricultural University, Hefei, China

**Keywords:** *Dendrobium officinale*, terpenoid, WGCNA, transcription factors, terpene synthase gene

## Abstract

*Dendrobium officinale* is a kind of traditional Chinese herbal medicine. Its flowers could be used as health care tea for its aroma flavor and medicinal value. Most recent studies demonstrated that terpenoids are the main components of the aromatic compounds in the flowers, but the biosynthesis of terpenoids is poorly understood in *D. officinale*. In the experiment, the flowers from two cultivars of *D. officinale* with different smells were collected. The transcriptome analysis and combined volatile terpenoids determination were performed to identify the genes related to the biosynthesis of the terpenoids. The results showed that the different products of volatile terpenoids are α-thujene, linalool, α-terpineol, α-phellandrene, γ-muurolene, α-patchoulene, and δ-elemene in two cultivar flowers. The transcriptome analysis detected 25,484 genes in the flowers. And 18,650 differentially expressed genes were identified between the two cultivars. Of these genes, 253 genes were mapped to the terpenoid metabolism pathway. Among these genes, 13 terpene synthase (TPS) genes may have correlations with AP2/ERF, WRKY, MYB, bHLH, and bZIP transcription factors by weighted gene co-expression network analysis (WGCNA). The transcription factors have regulatory effects on TPS genes. These results may provide ideas for the terpenoid biosynthesis and regulatory network of *D. officinale* flowers.

## Introduction

Terpenoids are a class of highly diverse natural products. There are more than 80,000 known terpenoids, and at least half of them are synthesized by plants (Nagegowda and Gupta, 2020). Terpenoids have diverse biological functions in nature. It plays an essential role in the interaction and mediation of antagonism between organisms (Abbas et al., 2017). It can be used to attract pollinators, defend against ground and underground herbivores, and transmit signals between plants (Raguso, 2008; Ali et al., 2012; Abbas et al., 2017). Otherwise, it can be used as natural flavors and aroma compounds, which have beneficial effects on human health (Wagner and Elmadfa, 2003). Volatile terpenoids found in almost all plant organs are the main part of volatile compounds in flowers (Das et al., 2013). The common volatile terpenes in orchid flowers are limonene, pinene, myrcene, linalool, and ocimene (Gao et al., 2018; Baek et al., 2019; Robustelli Della Cuna et al., 2019).

Terpenoids are synthesized from two common five-carbon precursors, isopentenyl pyrophosphate (IPP) and dimethylallyl pyrophosphate (DMAPP) (David, 2008). In plants, the mevalonate pathway (MVA pathway) and the methyl-D-erythritol-4-phosphate pathway (MEP pathway) are responsible for forming these C5-isoprene building blocks. The MVA pathway produces volatile sesquiterpenes (C_15_), while the MEP pathway provides precursors for volatile sesquiterpenes (C_5_), monoterpenes (C_10_), and diterpenes (C_20_) (Karunanithi and Zerbe, 2019). The MVA pathway consists of six enzymatic reactions. Three molecules of acetyl-CoA are gradually condensed into 3-hydroxy-3-methylglutaryl-CoA and then reduced to MVA, followed by two phosphorylation and decarboxylation reactions. Elimination eventually forms IPP (Lange et al., 2000). The MEP pathway involves seven enzymatic steps. It starts with the condensation of D-glycerol 3-phosphate (GAP) and pyruvate (Pyr) to form 1-deoxy-D-xylulose 5-Phosphoric acid, then undergoes isomerization/reduction to generate MEP.

Terpene synthase (TPS) is a key enzyme for terpene synthesis, which has been identified in *Arabidopsis*, *Malus domestica*, *and Solanum lycopersicum* (Aubourg et al., 2002; Nieuwenhuizen et al., 2013; Zhou and Pichersky, 2020). According to the different products formed, it can be classified as monoterpene synthase, sesquiterpene synthase, diterpene synthase, and so on. They are, respectively, geranyl pyrophosphate (GPP) or neryl pyrophosphate (NPP), farnesyl pyrophosphate (FPP), and geranylgeranyl pyrophosphate (GGPP) as precursor substrates to synthesize corresponding monoterpenes, sesquiterpenes, and diterpenes (Schilmiller et al., 2009). Besides, a variety of transcription factors (TFs) have been involved in the regulation of terpenoid biosynthesis, such as WRKY, bHLH, MYB, bZIP (Zhang et al., 2015), and AP2/ERF (Suttipanta et al., 2011; Lu et al., 2013; Zhang et al., 2015; Alex et al., 2016; Jiang et al., 2016; Paul et al., 2017; Pu et al., 2018; Majid et al., 2019). For example, *PbbHLH4* can induce high expression level of TPS genes in *Phalaenopsis bellina* (Orchidaceae) (Chuang et al., 2018). *Wintersweet* (*Chimonanthus praecox* L.) *CpMYC2* and *CpbHLH13* TFs may be involved in the positive regulation of biosynthesis of monoterpenes (linalool) and sesquiterpenes (β-caryophyllene) in transgenic plants of Wintersweet (*C. praecox* L.) (Ji et al., 2014). Overexpression of this gene can upregulate the transcript level of terpenoid-related genes in transient transformed *Conyza blinii* leaves (Sun et al., 2018).

*Dendrobium officinale* Kimura et Migo is a valuable Chinese traditional medicine for its beneficial effects, including anti-tumor, anti-angiogenesis, immune enhancement, anti-oxidation, and alleviating diabetes (Tang et al., 2017; Teixeira da Silva and Ng, 2017). Previous studies have identified 34 TPS genes in *D. officinale*. They were classified into four subfamilies (TPS-a, TPS-b, TPS-c, and TPS-e/f) (Yu et al., 2020). In the experiment, the volatile terpenoids in flowers were identified and quantitatively analyzed for two cultivars of Wanhu No.5 and Wanhu No.6 of *D. officinale*. By using transcriptome sequence and weighted gene co-expression network analysis (WGCNA), the terpene synthesis genes were identified. And the correlation between volatile terpenoids and differential gene expression levels was analyzed. The relationship between TFs AP2/ERF, WRKY, MYB, bHLH, and bZIP and terpenoid metabolism was explored.

## Materials and Methods

### Plant Material

Two cultivars of flowers, Wanhu No.5 and Wanhu No.6, came from the laboratory of Professor Cai Yongping of Anhui Agricultural University. The two cultivars of flowers were sampled at the flowering period in the full bloom period. A total of 12 flower samples were collected. There were three replicates for each of the two types of flowers, for terpenoid metabolic profiling and transcriptome analysis. To analyze the natural volatile compounds, the flowers of Wanhu No.5 and Wanhu No.6 were enclosed and sampled in an extraction bottle. Each cultivar of flowers was sealed into solid-phase microextraction (SPME) vials immediately for further analysis (Sun et al., 2016).

To investigate the spatiotemporal correlation between the TFs related to TPS and the emission of volatile terpene compounds, a range of samples, including two cultivars of flowers, were collected for RNA extraction. All samples were immediately frozen in liquid nitrogen and stored at –80°C until required.

### Gas Chromatography-Mass Spectrometry Analysis of Volatile Compounds in Flowers of Wanhu No.5 and Wanhu No.6

Headspace SPME was employed to collect the volatile compounds from flower tissues, which were absorbed by a 75 μm CAR/PDMS fiber (Sigma-Aldrich) for 2 h at 25°C. Total trapped volatile compounds were subsequently thermally desorbed and transferred to an Agilent 5975-6890N gas chromatography-mass spectrometry (GC-MS) apparatus (Agilent Technologies) equipped with HP5-MS quartz capillary column (250 μm diameter, 60 m length, and 0.25 μm film thickness). The instrument used for the gas chromatography–mass spectrometry analysis was an Agilent 7890B-7000B triple quadrupole gas-mass spectrometer. The carrier gas was helium with 1 ml/min of flow rate. The temperature of the electron ionization (EI) ion source is 230°C, and the electron energy was 70 eV. The temperature of the quadrupole is 150°C for 280°C of interface temperature, and the mass scanning range was 50–400 amu. The temperature program was isothermal at 60°C for 3 min, then increased at a rate of 5°C min^–1^ to 300°C, and was then mainteined at 300°C for 5 min.

Through the NIST (National Institute of Standards and Technology) 2011 standard library, the volatile compounds detected during the experiment were identified and qualitatively analyzed. The obtained compounds were compared with the literature to obtain the determined volatile terpenoids.

### Transcriptome Data Analysis

The Illumina NEBNext^®^ Ultra^TM^ RNA Library Prep Kit was used in the library construction. AMPure XP beads were used to screen 200 bp cDNA, and polymerase chain reaction (PCR) amplification was performed. AMPure XP beads were used to purify the PCR products again, and the library was finally obtained. RNA quality was evaluated on a NanoPhotometer^®^ spectrophotometer (IMPLEN, München, Germany). RNA-Seq was performed by LC-bio (Hangzhou, China) on the Illumina HiSeq 4000 platform. Raw reads obtained from RNA-Seq were pre-processed; adapters were trimmed, and low-quality and shorter reads were removed. The *Dendrobium* genome was selected as a reference for Wanhu No.5 and Wanhu No.6. Clean reads were aligned to the *Dendrobium* genome, and the GenBank assembly accession was GCA_001605985.2 (latest). The transcriptome data could be obtained on the National Center for Biotechnology Information (NCBI); the BioProject accession number was PRJNA703321. Q20, Q30, and GC contents of the clean data were calculated. Then the fragments per kilobase million (FPKM) of each gene was calculated based on the length of the gene, and the reads mapped to that gene were calculated (Bray et al., 2016).

The Kyoto Encyclopedia of Genes and Genomes (KEGG) is a database resource for understanding the advanced functions and utilities of biological systems, such as cells, organisms, and ecosystems, from molecular-level information, especially large-scale molecular data sets generated by genome sequencing and other high-throughput databases (Kanehisa et al., 2017). We used clusterProfiler R software to analyze the statistical enrichment of differentially expressed genes (DEGs) in the KEGG pathway. To map the target genes to metabolic pathways, all sequences of DEGs were uploaded to the Mercator v.3.6^1^ to generate a root map file, then it was imported to the Mapman software (V3.6.0 RC1) to obtain the map. In detail, the DEGs in the terpernoids biosynthesis pathway were displayed with the value of log2.Fold_change between Wanhu No.5 and Wanhu No.6.

### Gene Co-expression Analysis

We performed WGCNA on all DEGs screened out in transcriptome sequencing (Langfelder and Horvath, 2008). We used the WGCNA package to run the FPKM expression of DEGs in the R software. It had a module with default settings, the power was 6, minModuleSize was 30, and the minimum height of the combined module was 0.25. The gene with the highest connectivity within the module was called the intra-mold hub gene. Networks were visualized by Cytoscape software v 3.6.1 (Shannon et al., 2003).

### Quantitative Real-Time PCR (qRT-PCR) Assays

RNAprep pure plant kit (Biofit, Chengdu, China) was used to isolate total RNA from fresh Wanhu No.5 and Wanhu No.6 flowers (100 mg). According to the manufacturer’s instructions, RNA (1 μg) was used to synthesize cDNA using PrimeScript^TM^ RT kit with gDNA eraser (January, Perfect Real Time, Takara, Tokyo, Japan). Using QuantStudio 6 Flex real-time PCR system (Thermo Fisher, Waltham, MA, United States) and SYBR^®^ PremixExTaq^TM^II (2x) (Japan, Takara), the gene expression level was detected by qRT-PCR. We used NCBI-BLAST online software^2^ to design fluorescent quantitative primers for the key genes in the terpene synthesis pathway (Supplementary Table 1). The results are attached in Figure 11. The reaction steps are 50°C 2 min, 95°C 30 s, 95°C 5 s, 60°C 34 s, 40 cycles, and 72°C for 10 min (Jin et al., 2013). The 2^–ΔΔ*CT*^ method was used to calculate the relative gene expression, and the experiment was repeated three times (Livak and Schmittgen, 2001).

### Statistical Analysis

Average and standard derivations of chemicals were calculated using Microsoft Excel software. The results of GC-MS were drawn using Origin 6.0 (Origin Lab Corporation, United States). Prism 8 was used for some figures. R software was used to calculate correlation factors. The phylogenetic analysis included the genomes of *D. officinale*. The protein sequences were aligned using the default parameters in MUSCLE^3^, and then the neighbor-joining tree was generated by guided analysis (1,000 repeats) using MEGA 7.0 software (Fan et al., 2020).

## Results

### GC-MS Analysis of Volatile Terpenoids in Flowers of Wanhu No.5 and Wanhu No.6

**FIGURE 1:**
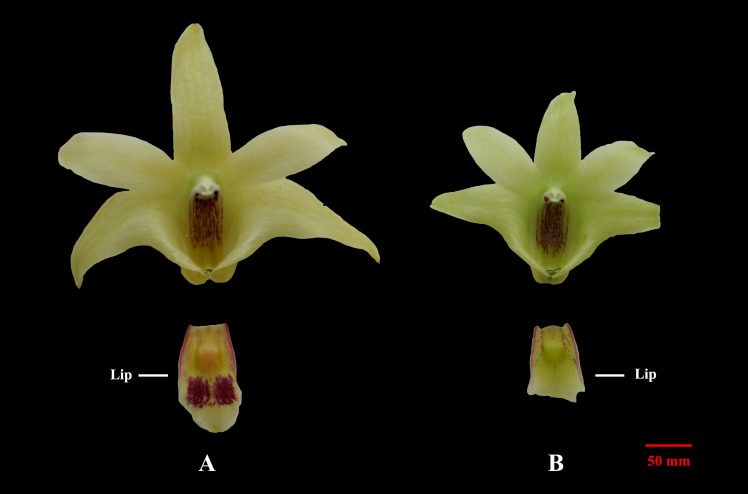
Two cultivars of *Dendrobium officinale* flowers. **(A)** Wanhu No.5. **(B)** Wanhu No.6.

**FIGURE 2:**
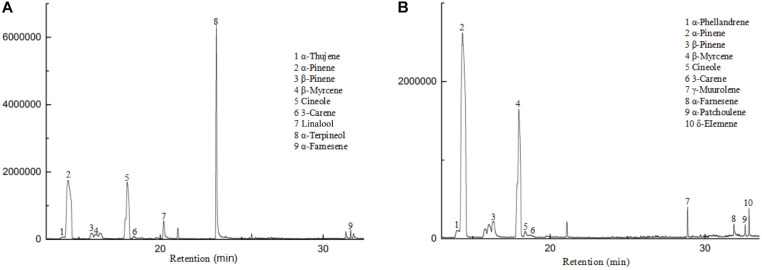
GC-MS analysis of the products formed in two cultivars of *Dendrobium officinale* flowers. **(A)** Wanhu No.5. **(B)** Wanhu No.6.

**FIGURE 3:**
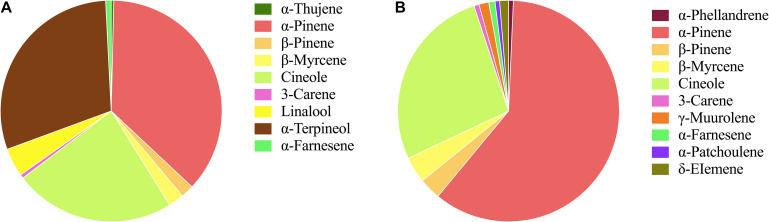
Volatile terpenoids compounds of two cultivars of *Dendrobium officinale* flowers. **(A)** Wanhu No.5. **(B)** Wanhu No.6. The same color represents the same terpenoid.

The flowers from two cultivars, Wanhu No.5 and Wanhu No.6 of *D. officinale*, were collected for GC-MS analysis (Figure 1). The results showed that the compositions of volatile compounds in the two cultivars differed from each other. There were 18 volatile compounds detected in flowers of Wanhu No.5, which contained 80% terpenoids and 8.6% alkane compounds. Otherwise, 20 volatile compounds were detected in flowers of Wanhu No.6. The ratios of terpenes and alkanes accounted for 84.57 and 5.51%, respectively. Among these detected volatile terpenoids, a total of 13 volatile terpenoids were detected in the two cultivars. Of these components, nine volatile terpenoids were detected in Wanhu No.5, mainly including α-pinene (36.63%), cineole (23.51%), α-terpineol (29.81%), linalool (4.18%), β-myrcene (2.26%), and β-pinene (1.89%) (Figure 2A). Ten volatile terpenoids were detected in Wanhu No.6. The main terpenoids were α-pinene (60.29%), cineole (26.96%), β-myrcene (3.83%), β-pinene (3.17%), γ-muurolene (1.43%), and δ-elemene (1.3%) (Figure 2B). So volatile terpenoids’ content and compositions in the two cultivars, Wanhu No.5 and Wanhu No.6, differed from each other. α-Thujene, linalool, and α-terpineol were only detected in Wanhu No.5, and six volatile terpenoids, such as α-phellandrene, γ-muurolene, α-patchoulene, and δ-elemene, were only detected in Wanhu No.6. The volatile terpenoids content was higher in Wanhu No.6 than in Wanhu No.5 (Figure 3).

### RNA-Seq and DEGs Analysis

**FIGURE 4:**
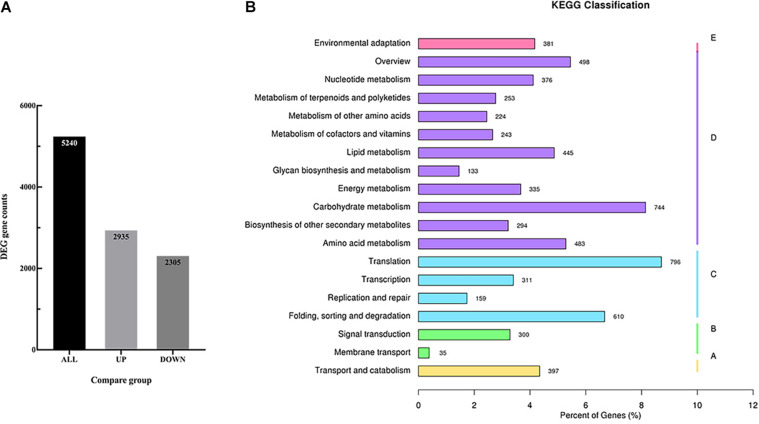
RNA-seq and different expression genes (DEGs) analysis. **(A)** Statistical histogram of the number of different genes in the different comparison combinations. The numbers on the columns indicate the number of differential genes. **(B)** KEGG pathway classification map of DEGs.

In order to identify the candidate genes related to the volatile terpenoids biosynthesis, RNA-seq was performed using the flowers. As a result, a total of 25,484 genes were detected and mapped to the *D. officinale* genome. Of these genes, 5,240 genes were identified with differentially expressed levels between Wanhu No.5 and Wanhu No.6 (Figure 4A). If compared with Wanhu No.5, 2,935 genes were upregulated, and 2,305 genes were downregulated in Wanhu No.6. And KEGG cluster analysis showed 253 unigenes involved in the metabolism of terpenoids and polyketides pathway (Figure 4B).

**FIGURE 5:**
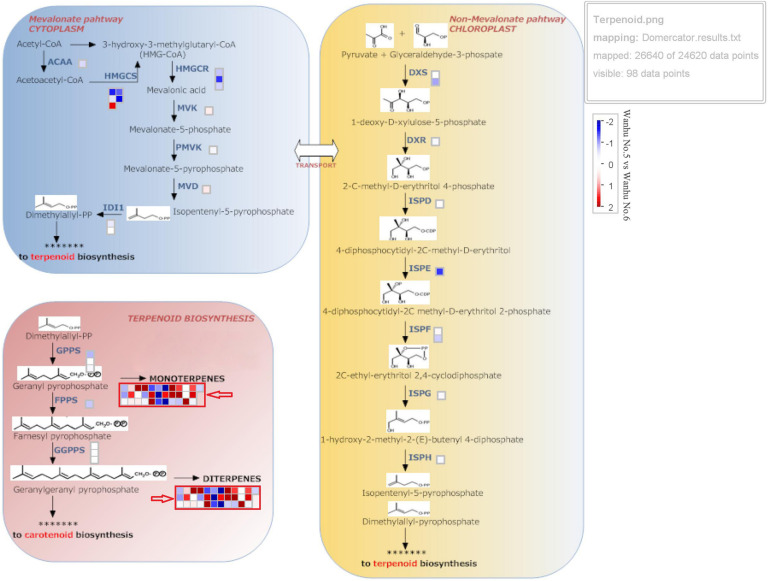
Expression pattern of genes related to terpenoid biosynthesis. Gene expression levels in the fully opened flowers of Wanhu No.5 and Wanhu No.6 are represented by color gradations. AACT, acetyl-CoA acetyltransferase; CMK, 4-(cytidine 59-diphospho)-2-C-methyl-D-erythritol kinase; DXR, 1-deoxy-D-xylulose 5-phosphate reductoisomerase; DXS, DXP synthase; FPPS, farnesyl diphosphate synthase; GPPS, geranyl diphosphate synthase; HDS, 4-hydroxy-3-methyl-2-butenyl pyrophosphate synthase; HMGR, HMG-CoA reductase; HMGS, HMG-CoA synthase; IDI, isopentenyl diphosphate isomerase; IDS, isoprenyl diphosphate synthase; MCT, 2-C-methyl-D-erythritol 4-phosphate cytidylyltransferase; ME, ME-CDP synthase; MPDC, mevalonate diphosphate decarboxylase; MVK, mevalonate kinase; PMK, phosphomevalonate kinase; TPS, terpene synthase.

Terpenoids are synthesized through the MVA and methylerythritol phosphate (MEP) pathways, which are independent but complementary to each other. The MEP pathway is mainly responsible for the biosynthesis of monoterpenoids, accounting for about 53% of the total terpenoids in flowers; sesquiterpenes are synthesized through the MVA pathway, accounting for about 28% of the total terpenoids in flowers. Figure 5 shows that TPS in *D. officinale* Wanhu No.5 was quite different from that in Wanhu No.6, and the number of upregulated genes was greater than that of downregulated genes. The squares in red frames marked with red arrows indicate the differentially expressed TPS genes between Wanhu No.6 and Wanhu No.5. The red squares reveal the upregulated genes in Wanhu No.5 compared with those in Wanhu No.6. The blue frames showed the downregulated genes in Wanhu No.5 in contrast with Wanhu No.6. In the MEP pathway, two genes, HMGR and HMGS, were expressed differently in Wanhu No.5 and Wanhu No.6, which was also the main reason for the different terpenes produced by the two flowers.

### Analysis of Gene Correlation Network

**FIGURE 6:**
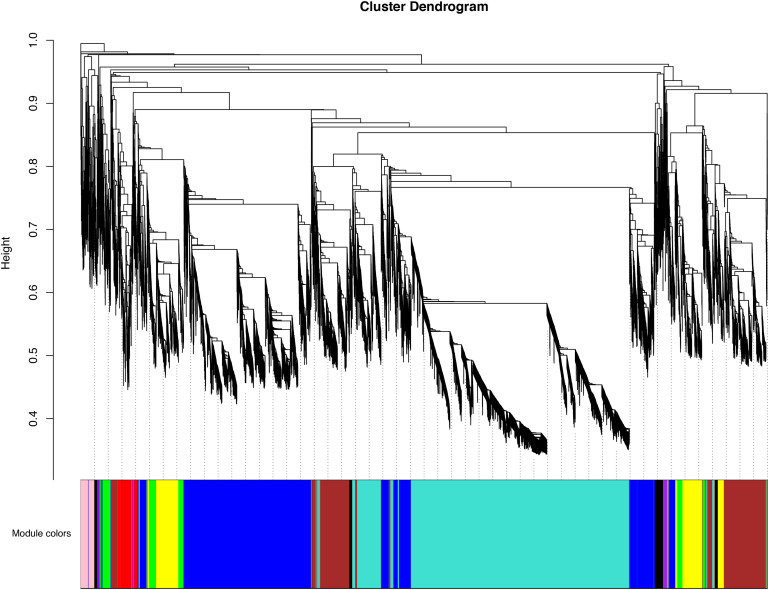
Clustering dendrogram of expressed genes. Gene modules were identified by dynamic hierarchical tree cut and shown in different colors. Height cut = 0.25, minimal module size = 30.

In order to obtain the comprehensive transcriptome changes of two cultivars of *D. officinale* flowers, we established a weighted gene co-expression network to classify 18650 DEGs. Genes that were more closely related to each other would be gathered in the same module, and finally 10 modules would be obtained (Figure 6). The biggest module was the turquoise module, which contains 6,047 genes. However, the smallest module contained 6 genes. The expression patterns of the different modules of the two cultivars of flowers were also quite different from each other. For Wanhu No.5, the turquoise module’s gene expression was higher, while for Wanhu No.6 the gene expression of the blue module was higher (Supplementary Figure 1).

**FIGURE 7:**
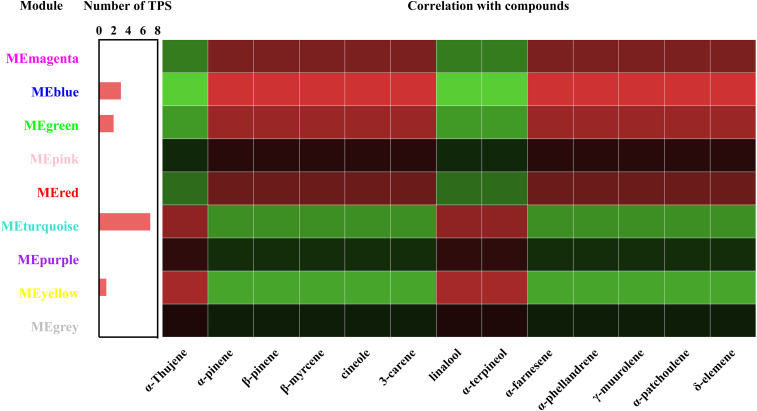
Correlation coefficients between terpene synthase (TPS), module, and volatile compound. Each row represents a module, and each column represents a terpenoid. The color of each block at the row–column intersection indicates the correlation coefficient: red for high positive correlation and green for high negative correlation, with a scale shown on the right of the panel.

To understand the relationship between terpenoids and genes, a relationship module diagram between genes and volatile terpenoids was analyzed. As was shown in Figure 7, the correlations between different modules and volatile terpenoids were relatively close, except for thujene, linalool, and terpineol, which were the different metabolites in Wanhu No.5 and Wanhu No.6. After screening, we found that the module contains 13 TPS genes, including 7 TPS genes in the turquoise module, 3 TPS genes in the blue module, 2 TPS genes in the green module, and 1 TPS gene in the yellow module. Different TPS genes might be closely related to the production of these volatile terpenoids through the relationship diagram.

**FIGURE 8:**
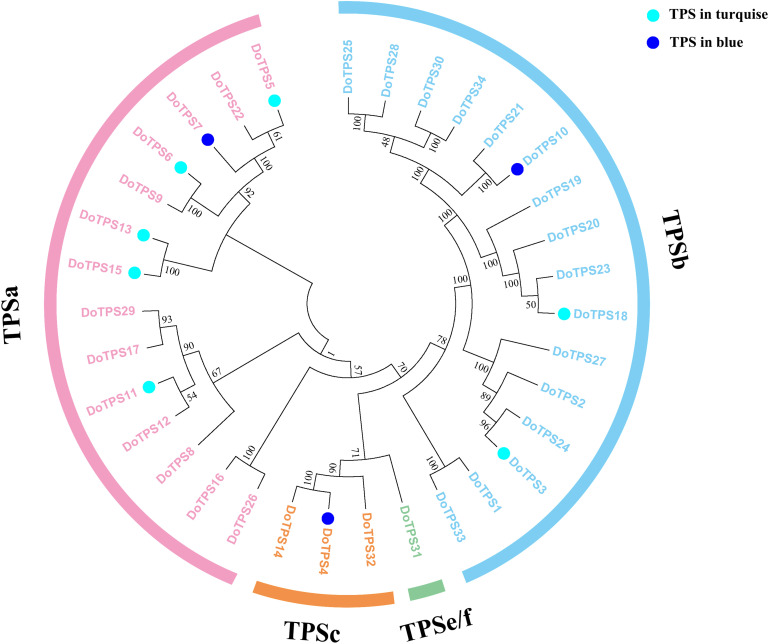
Phylogeny tree analysis of *Dendrobium officinale* TPSs. Triangles represent finger citron TPSs, and filled colors indicate their location in modules shown in Figure 6. Sequence alignment was performed by ClustalX. Phylogeny tree was visualized by MEGA7.

To further clarify TPS genes’s potential roles in the turquoise module and the blue module, we generated a phylogenetic tree by neighbor-joining method (Yu et al., 2020). Figure 8 showed that *DoTPSs* proteins were classified into four different clades, 14 in TPS-a, 16 in TPS-b, 3 in TPS-c, and 1 in TPS-e/f. The function of TPS-a family was to synthesize sesquiterpene synthase. TPS-b mainly synthesized monoterpene synthase and isoprene synthase. The function of TPS-c family was to synthesize the bifunctional class I/II (terephthaloyl diphosphate synthase/kaurene) involved in secondary metabolism, and the monofunctional class II included diterpene synthase (terephthaloyl diphosphate synthase enzyme) and diterpene synthase. TPS-e/f was a monofunctional class I diterpene synthase, diterpene synthase, sesquiterpene synthase, and monoterpene synthase involved in secondary metabolism (Alicandri et al., 2020). The phylogenetic tree showed that *DoTPS* in the blue module, including *DoTPS04*, *DoTPS10*, and *DoTPS07*, were located in TPS-a, TPS-b, and TPS-c; *DoTPS* in the turquoise module, including *DoTPS13*, *DoTPS03*, *DoTPS05*, *DoTPS06*, *DoTPS11*, *DoTPS15*, and *DoTPS18*, were located in TPS-a and TPS-b.

### TFs Regulating Terpenoid Synthases in Two *D. officinale* Cultivars

As listed in Supplementary Tables 3–8, the gene IDs and relative transcript levels of five TF families were obtained from the transcriptomes data of the two cultivars of *D. officinale* flowers. As is shown in Supplementary Figures 2–6, the expression patterns of TFs and *DoTPSs* in Wanhu No.5 were different from those in Wanhu No.6. The relative transcript levels were higher in Wanhu No.5. Also, the differences between Wanhu No.5 and Wanhu No.6 were significant in Supplementary Figures 3–8. And 10 terpenoid synthases and their read counts were collected in the transcriptome. When compared with Wanhu No.6, six TPS genes were upregulated with more than 2-fold (>2-fold), and eight TPS genes were downregulated (<–2-fold).

**FIGURE 9:**
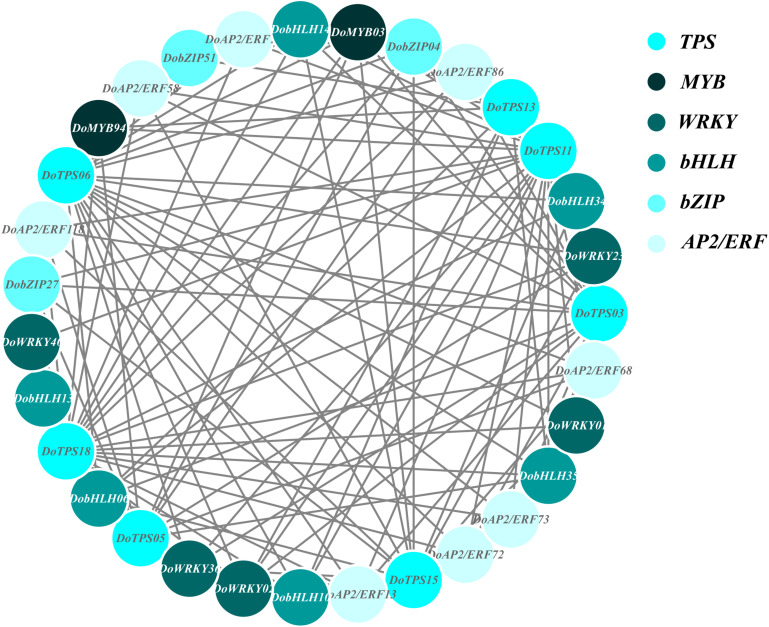
Gene co-expression subnetwork of the turquoise module. Network was reconstructed by edge weight cutoff = 0.25 and visualized by Cytoscape.

**FIGURE 10:**
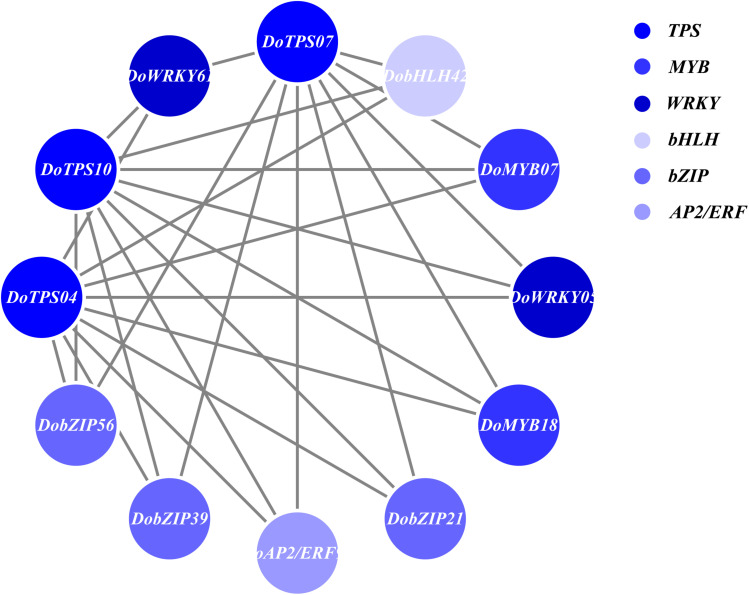
Gene co-expression subnetwork of the blue module. Network was reconstructed by edge weight cutoff = 0.25 and visualized by Cytoscape.

In order to understand the relationship between TPS genes and TFs, 12 WRKY, 12 bHLH, 9 MYB, 9 bZIP, and 14 AP2/ERF were selected for the following analysis. Seven TPS genes, *DoTPS13*, *DoTPS03*, *DoTPS05*, *DoTPS06*, *DoTPS11*, *DoTPS15*, and *DoTPS18*, were used based on the WGCNA analysis outcome (Figure 9). As a result, WRKY (*DoWRKY02*, DoWRKY01, *DoWRKY36*, *DoWRKY40*, *DoWRKY23*), bHLH (*DobHLH06*, *DobHLH10*, *DobHLH13*, *DobHLH14*, *DobHLH34*, *DobHLH35*), and AP2/ERF (*DoAP2/ERF19*, *DoAP2/ERF19*, *DoAP2/ERF68*, *DoAP2/ERF72*, *DoAP2/ERF86*, *DoAP2/ERF118*) genes were predicted to be closely related to TPS genes. TFs *DoWRKY05*, *DoWRKY61*, *DoMYB07*, *DoMYB18*, *DobHLH42*, *DobZIP39*, *DobZIP56*, *DobZIP21*, and *DoAP2/ERF90* were probably involved in the regulation of *DoTPS04*, *DoTPS10*, and *DoTPS07* (Figure 10).

### Verification of Gene Expression

**FIGURE 11:**
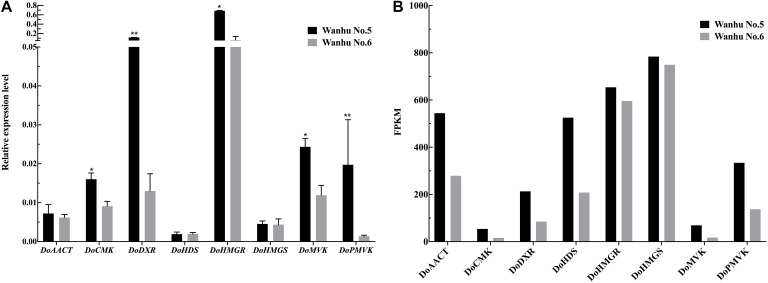
Expression patterns of eight genes as verified by qRT-PCR. **(A)** The relative expression levels of Wanhu No.5 and Wanhu No.6. **(B)** FPKM values from RNA-Seq. Values shown are mean ± SE of three replicates. “*” indicates that the difference is significant, “**” indicates that the difference is very significant.

In order to verify the transcriptome data, terpenoid synthesis pathway genes and related TFs were selected for real-time PCR analysis (Figure 11). The results showed that the expression patterns of the selected genes were consistent with the transcriptome data.

## Discussion

The flowers of *D. officinale* not only have a certain ornamental value but are also a kind of Chinese medicine that can be used to make tea with a certain anti-cancer effect (Lai, 2020). In *Dendrobium chrysanthum*, some terpenes including α-phellandrene, α-pinene, α-thujene, L- β-myrcene, α-terpinene, O-cymene, D-limoene, β-ocimene, and carene were detected in the flowers. The volatile components of *Dendrobium lohohense* flowers are mainly esters, and the aroma composition of *Dendrobium densiflorum* is mainly alkanes. The volatile components of *Dendrobium hancockii* and *D. officinale* are mainly terpenes (Li et al., 2015; Lv et al., 2016). In the experiment, 18 and 20 volatile compounds were detected from Wanhu No.5 and Wanhu No.6 flowers, respectively. There were 9 volatile terpenoids compounds detected in Wanhu No.5, and 10 in Wanhu No.6. α-Pinene is the most abundant compound in the flowers of the two cultivars. The volatile terpenoids are quite different in the two cultivars of *D. officinale*. For example, α-thujene, linalool, and α-terpineol were only detected in Wanhu No.5, while α-phellandrene, γ-muurolene, α-patchoulene, and δ-elemene were only found in Wanhu No.6.

The comprehensive analysis of gene co-expression and terpenoid accumulation has recently provided new insights into the regulation of terpenoid metabolism (Tai et al., 2018). In order to understand the regulation of terpenoid biosynthesis in *D. officinale*, the expressed genes detected by RNA-Seq were connected by using the method of WGCNA, which provides a network of nodes (genes) and edges (connections). This method is mainly about obtaining connections in the network based on gene co-expression data. This strategy has been used to discover potential target genes and TFs in plants (Ferreira et al., 2016). We obtained 10 different expression modules after WGCNA analysis of the transcriptome data. The results can provide a way to build a network of mining potential target genes and TFs in plants. In *D. officinale*, with 34 TPS genes, 13 TPS genes were obtained in WGCNA analysis. The function of *DoTPS10* has been verified; located in chloroplasts, *DoTPS10* uniquely converted geranyl diphosphate to linalool *in vitro* (Zhao et al., 2020).

In the present study, the top two modules enriched for TPS genes were selected for further analysis. Based on the WGCNA analysis, we obtained 13 TPS genes and 5 kinds of TFs that are related to the synthesis of TPS: AP2/ERF, bHLH, MYB, WRKY, and bZIP (Lu et al., 2013; Zhang et al., 2015; Pu et al., 2018; Majid et al., 2019). Through the correlation network diagram, we found that different TFs regulate different TPS differently. Transient expression of *AaERF1* and *AaERF2* can increase the transcription of amorpha-4,11-diene synthase (ADS) and *CYP71AV1* and increase accumulation of artemisinin and artemisinic acid (Sun et al., 2018). The *Arabidopsis MYC2* TF could bind to the promoter regions of the *TPS21* and *TPS11* that catalyzed sesquiterpenes’ formation to activate their expression, thereby increasing the release of sesquiterpenes (Hong et al., 2012). A peltate glandular trichomes (PGT)-specific R2R3-MYB gene, *MsMYB*, was identified in the RNA-Seq comparison data in spearmint. The analysis of the transgenic lines showed increased levels of monoterpenes. In contrast, the levels of *MsMYB* overexpression lines decreased (Reddy et al., 2017). In *Catharanthus roseus*, overexpression of *Cr-WRKY1* could downregulate the expression levels of *ORCA2/3*, *CrMYC2*, and zinc-finger *C. roseus* transcription factors (ZCTs) to regulate the synthesis of monoterpenes (Suttipanta et al., 2011). *AaAPK1* interacted with *AabZIP1* in *Artemisia annua*, and *AaAPK1* enhanced the transactivation activity of *AabZIP1* on artemisinin biosynthesis genes through phosphorylation (Zhang et al., 2018). Regarding the TPS genes identified based on WGCNA, there are eight in the TPS-a family, four in the TPS-b family, and one in the TPS-c family. TPS-a members are related to sesquiterpene formation, and TPS-b is related to monoterpene biosynthesis (Alicandri et al., 2020). Therefore, these identified TPSs might play an important role in producing volatile terpenoids in *D. officinale*. As the last enzymatic step of the MVA and MEP pathways, TPS is responsible for the direct synthesis of terpenoids (Schilmiller et al., 2009). However, in most cases, the expression level of these TPS genes has no linear relationship with their product content. There are two main reasons for uncertainty (Degenhardt et al., 2009). Firstly, a considerable amount of TPS was a multi-product enzyme that can produce multiple volatiles from a single substrate. Secondly, the replication and evolution of the TPS family produced isozymes that express different functions in time and space (Takehiko et al., 2014). There were too few studies on the TPS gene of *D. officinale*, so more experiments are needed to test the functions of these 13 *DoTPS*, such as overexpression in *Escherichia coli* for *in vitro* enzymatic analysis and stable transformation for *in vivo* functional analysis (Zhou and Pichersky, 2020). The regulation between *DoTPS* and TFs will be detected in more experiments.

## Conclusion

The transcriptome analysis in the two cultivars of *D. officinale* with differences in volatile terpenoid products was performed in order to mine the biosynthetic pathway related genes and regulatory mechanisms of the terpenoid metabolites of *D. officinale*. In the analysis of the two cultivars of *D. officinale* transcriptomes, the expression of upstream genes in the MVA and MEP pathways did not change much, and the TPS genes were quite different. Therefore, the diversity of terpenoids was caused by the differential expression of TPS. We obtained 10 gene modules from WGCNA. From the gene module, we screened 13 TPS genes and AP2/ERF, WRKY, MYB, bHLH, and bZIP TFs, analyzed the correlation between these TFs and TPS expression, and found that these TFs were displayed in the position of the correlation network. They played a role in regulating terpenoid metabolism. Future work should focus on the direct and indirect interactions between TPS and related TFs to clarify the functional network that controls terpene production. These results might provide ideas for the terpenoid biosynthesis and regulatory network of *D. officinale* flowers.

## Data Availability Statement

The datasets presented in this study can be found in online repositories. The names of the repository/repositories and accession number(s) can be found below: National Center for Biotechnology Information (NCBI) BioProject, https://www.ncbi.nlm.nih.gov/bioproject/, PRJNA703321.

## Author Contributions

HF designed the study. NL wrote the manuscript. NL, YD, and ML performed the experiments. XS, LL, and LQ helped in data analysis and manuscript preparation. YC revised the manuscript. All authors contributed to the article and approved the submitted version.

## Conflict of Interest

The authors declare that the research was conducted in the absence of any commercial or financial relationships that could be construed as a potential conflict of interest.

## Footnotes

1 https://www.plabipd.de/portal/web/guest/mercator-sequence-annotation

2 https://www.ncbi.nlm.nih.gov/tools/primer-blast/index.cgi?LINK_LOC=BlastHome

3 https://www.ebi.ac.uk/Tools/msa/muscle/

## Supplementary Material

Supplementary Figure 1 Functional annotation of unigenes from *D. officinale* flower transcriptome against databases. **(A)** Venn diagram showing the number of unigenes annotated in two cultivars of *D. officinale* flowers. **(B)** The cluster of transcriptome gene Wanhu No.5 and Wanhu No.6.

Supplementary Figure 2 Hierarchical cluster tree showing 10 modules of co-expressed genes.

Supplementary Figure 3 Heatmap about *DoTPS* of two cultivars of *D. officinale* flower.

Supplementary Figure 4 Heatmap about *DobHLH* of two cultivars of *D. officinale* flower.

Supplementary Figure 5 Heatmap about *DobZIP* of two cultivars of *D. officinale* flower.

Supplementary Figure 6 Heatmap about *DoAP2/ERF* of two cultivars of *D. officinale* flower.

Supplementary Figure 7 Heatmap about *DoWRKY* of two cultivars of *D. officinale* flower.

Supplementary Figure 8 Heatmap about *DoMYB* of two cultivars of D. officinale flower.

Supplementary Table 1 Primers used for RT-qPCR analysis.

Supplementary Table 2 Gene IDs and relative transcript levels of *DobZIP* of two cultivals of *D. officinale* flower.

Supplementary Table 3 Gene IDs and relative transcript levels of *DoMYB* of two cultivals of *D. officinale* flower.

Supplementary Table 4 Gene IDs and relative transcript levels of *DoAP2/ERF* of two cultivals of *D. officinale* flower.

Supplementary Table 5 Gene IDs and relative transcript levels of *DoWRKY* of two cultivals of *D. officinale* flower.

Supplementary Table 6 Gene IDs and relative transcript levels of *DobHLH* of two cultivals of *D. officinale* flower.

## References

[B1] Abbas F. Ke Y. Yu R. Yue Y. Amanullah S. Jahangir M. M. (2017). Volatile terpenoids: multiple functions, biosynthesis, modulation and manipulation by genetic engineering. *Planta* 246 803–816. 28803364 10.1007/s00425-017-2749-x

[B2] Alex V. M. Priscille S. Ivo G. Javiera E. Fabian S. Karel M. (2016). The basic helix-loop-helix transcription factor BIS2 is essential for monoterpenoid indole alkaloid production in the medicinal plant *Catharanthus roseus*. *Plant J*. 88 3–12. 27342401 10.1111/tpj.13230

[B3] Ali J. G. Alborn H. T. Campos-Herrera R. Kaplan F. Duncan L. W. Rodriguez-Saona C. (2012). Subterranean, herbivore-induced plant volatile increases biological control activity of multiple beneficial nematode species in distinct habitats. *PLoS One* 7:e38146. 10.1371/journal.pone.0038146 22761668 PMC3384653

[B4] Alicandri E. Paolacci A. R. Osadolor S. Sorgona A. Badiani M. Ciaffi M. (2020). On the evolution and functional diversity of terpene synthases in the *Pinus* species: a review. *J. Mol. Evol*. 88 253–283. 10.1007/s00239-020-09930-8 32036402

[B5] Aubourg S. Lecharny A. Bohlmann J. (2002). Genomic analysis of the terpenoid synthase (AtTPS) gene family of *Arabidopsis thaliana*. *Mol. Genet. Genomics* 267 730–745. 10.1007/s00438-002-0709-y 12207221

[B6] Baek Y. S. Ramya M. An H. R. Park P. M. Lee S. Y. Baek N. I. (2019). Volatiles Profile of the floral organs of a new hybrid *Cymbidium*, ‘Sunny Bell’ using headspace solid-phase microextraction gas chromatography-mass spectrometry analysis. *Plants* 8:251. 10.3390/plants8080251 31357642 PMC6724120

[B7] Bray N. L. Pimentel H. Melsted P. Pachter L. (2016). Erratum: near-optimal probabilistic RNA-seq quantification. *Nat. Biotechnol*. 34:888. 10.1038/nbt0816-888d 27504780

[B8] Chuang Y. C. Hung Y. C. Tsai W. C. Chen W. H. Chen H. H. (2018). PbbHLH4 regulates floral monoterpene biosynthesis in *Phalaenopsis orchids*. *J. Exp. Bot*. 69 4363–4377. 10.1093/jxb/ery246 29982590 PMC6093345

[B9] Das A. Lee S. H. Hyun T. K. Kim S. W. Kim J. Y. (2013). Plant volatiles as method of communication. *Plant Biotechnol. Rep*. 7 9–26.

[B10] David W. C. (2008). Unearthing the roots of the terpenome. *Curr. Opin. Chem. Biol*. 12 141–150. 18249199 10.1016/j.cbpa.2007.12.008 PMC2430190

[B11] Degenhardt J. Köllner T. G. Gershenzon J. (2009). Monoterpene and sesquiterpene synthases and the origin of terpene skeletal diversity in plants. *Phytochemistry* 70 1621–1637. 19793600 10.1016/j.phytochem.2009.07.030

[B12] Fan H. Cui M. Li N. Li X. Liang Y. Liu L. (2020). Genome-wide identification and expression analyses of R2R3-MYB transcription factor genes from two orchid species. *PeerJ* 8:e9781. 10.7717/peerj.9781 32953268 PMC7473048

[B13] Ferreira S. S. Hotta C. T. Poelking V. G. Leite D. C. Buckeridge M. S. Loureiro M. E. (2016). Co-expression network analysis reveals transcription factors associated to cell wall biosynthesis in sugarcane. *Plant. Mol. Biol*. 91 15–35. 10.1007/s11103-016-0434-2 26820137 PMC4837222

[B14] Gao F. Liu B. Li M. Gao X. Fang Q. Liu C. (2018). Identification and characterization of terpene synthase genes accounting for volatile terpene emissions in flowers of *Freesia* x hybrida. *J. Exp. Bot*. 69 4249–4265. 10.1093/jxb/ery224 29901784 PMC6093421

[B15] Hong G. J. Xue X. Y. Mao Y. B. Wang L. J. Chen X. Y. (2012). Arabidopsis MYC2 interacts with DELLA proteins in regulating sesquiterpene synthase gene expression. *Plant Cell* 24 2635–2648. 10.1105/tpc.112.098749 22669881 PMC3406894

[B16] Ji Y. Xiao J. Shen Y. Ma D. Li Z. Pu G. (2014). Cloning and characterization of AabHLH1, a bHLH transcription factor that positively regulates artemisinin biosynthesis in *Artemisia annua*. *Plant Cell Physiol*. 55 1592–1604. 10.1093/pcp/pcu090 24969234

[B17] Jiang W. Fu X. Pan Q. Tang Y. Shen Q. Lv Z. (2016). Overexpression of AaWRKY1 leads to an enhanced content of artemisinin in *Artemisia annua*. *Biomed. Res. Int*. 2016:7314971. 10.1155/2016/7314971 27064403 PMC4809039

[B18] Jin Q. Yao Y. Cai Y. Lin Y. (2013). Molecular cloning and sequence analysis of a phenylalanine ammonia-lyase gene from *Dendrobium*. *PLoS One* 8:e62352. 10.1371/journal.pone.0062352 23638048 PMC3640076

[B19] Kanehisa M. Furumichi M. Tanabe M. Sato Y. Morishima K. (2017). KEGG: new perspectives on genomes, pathways, diseases and drugs. *Nucleic Acids Res*. 45 D353–D361. 10.1093/nar/gkw1092 27899662 PMC5210567

[B20] Karunanithi P. S. Zerbe P. (2019). Terpene synthases as metabolic gatekeepers in the evolution of plant terpenoid chemical diversity. *Front. Plant Sci*. 10:1166. 10.3389/fpls.2019.01166 31632418 PMC6779861

[B21] Lange B. M. Rujan T. Martin W. Croteau R. (2000). Isoprenoid biosynthesis: the evolution of two ancient and distinct pathways across genomes. *Proc. Natl. Acad. Sci*. 97 13172–13177. 10.1073/pnas.240454797 11078528 PMC27197

[B22] Langfelder P. Horvath S. (2008). WGCNA: an R package for weighted correlation network analysis. *BMC Bioinformatics* 9:559. 10.1186/1471-2105-9-559 19114008 PMC2631488

[B23] Lai Y. (2020). Study on the efficacy of *Dendrobium* flower. *Technol. Wind* 18:277. 10.19392/j.cnki.1671-7341.202018211

[B24] Li C. Mingzhong H. Shaohua H. Junmei Y. (2015). Volatile components in flowers of four *Dendrobium* species. *J. Trop. Subtrop. Bot.* 4, 454–462.

[B25] Livak K. J. Schmittgen D. T. (2001). Analysis of relative gene expression data using real-time quantitative PCR and the 2(-Delta Delta C(T)) method. *Methods* 25 402–408. 11846609 10.1006/meth.2001.1262

[B26] Lu X. Zhang L. Zhang F. Jiang W. Shen Q. Zhang L. (2013). AaORA, a trichome-specific AP2/ERF transcription factor of *Artemisia annua*, is a positive regulator in the artemisinin biosynthetic pathway and in disease resistance toBotrytis cinerea. *New Phytol*. 198 1191–1202. 23448426 10.1111/nph.12207

[B27] Lv S. Meng X. Xinfeng Z. Jinjing L. Jinping S. (2016). Studies on volatile constituents of 11 families of *Dendrobium* officinale flowers. *Chin. J. Exp. Tradit. Med. Formula.* 6, 52–57. 10.13422/j.cnki.syfjx.2016060052

[B28] Majid I. Kumar A. Abbas N. (2019). A basic helix loop helix transcription factor, AaMYC2-Like positively regulates artemisinin biosynthesis in *Artemisia annua* L. *Ind. Crops Prod*. 128 115–125.

[B29] Nagegowda D. A. Gupta P. (2020). Advances in biosynthesis, regulation, and metabolic engineering of plant specialized terpenoids. *Plant Sci*. 294: 110457. 10.1016/j.plantsci.2020.110457 32234216

[B30] Nieuwenhuizen N. J. Green S. A. Chen X. Bailleul E. J. Matich A. J. Wang M. Y. (2013). Functional genomics reveals that a compact terpene synthase gene family can account for terpene volatile production in apple. *Plant Physiol*. 161 787–804. 10.1104/pp.112.208249 23256150 PMC3561019

[B31] Paul P. Singh S. K. Patra B. Sui X. Pattanaik S. Yuan L. (2017). A differentially regulated AP 2/ERF transcription factor gene cluster acts downstream of a MAP kinase cascade to modulate terpenoid indole alkaloid biosynthesis in *Catharanthus roseus*. *New Phytol*. 213 1107–1123. 27801944 10.1111/nph.14252

[B32] Pu S. Xueqing F. Qian S. Meng L. Qifang P. Yueli T. (2018). The roles of AaMIXTA1 in regulating the initiation of glandular trichomes and cuticle biosynthesis in *Artemisia annua*. *New phytol*. 217 261–276. 28940606 10.1111/nph.14789

[B33] Raguso R. A. (2008). Wake up and smell the roses: the ecology and evolution of floral scent. *Annu. Rev. Ecol. Evol. Syst*. 39 549–569. 10.1146/annurev.ecolsys.38.091206.095601

[B34] Reddy V. A. Wang Q. Dhar N. Kumar N. Venkatesh P. N. Rajan C. (2017). Spearmint R2R3-MYB transcription factor MsMYB negatively regulates monoterpene production and suppresses the expression of geranyl diphosphate synthase large subunit (MsGPPS.LSU). *Plant Biotechnol. J*. 15 1105–1119. 10.1111/pbi.12701 28160379 PMC5552485

[B35] Robustelli Della Cuna F. S. Calevo J. Bari E. Giovannini A. Boselli C. Tava A. (2019). Characterization and antioxidant activity of essential oil of four sympatric orchid species. *Molecules* 24:3878. 10.3390/molecules24213878 31661846 PMC6864456

[B36] Schilmiller A. L. Schauvinhold I. Larson M. Xu R. Charbonneau A. L. Schmidt A. (2009). Monoterpenes in the glandular trichomes of tomato are synthesized from a neryl diphosphate precursor rather than geranyl diphosphate. *Proc. Natl. Acad. Sci. U.S.A*. 106 10865–10870. 10.1073/pnas.0904113106 19487664 PMC2705607

[B37] Shannon P. Markiel A. Ozier O. Baliga N. S. Wang J. T. Ramage D. (2003). Cytoscape: a software environment for integrated models of biomolecular interaction networks. *Genome Res*. 13 2498–2504. 10.1101/gr.1239303 14597658 PMC403769

[B38] Sun W. Liang L. Meng X. Li Y. Gao F. Liu X. (2016). Biochemical and molecular characterization of a flavonoid 3-O-glycosyltransferase responsible for anthocyanins and flavonols biosynthesis in *Freesia* hybrida. *Front. Plant Sci*. 7:410. 10.3389/fpls.2016.00410 27064818 PMC4815329

[B39] Sun W.-J. Zhan J.-Y. Zheng T.-R. Sun R. Wang T. Tang Z.-Z. (2018). The jasmonate-responsive transcription factor CbWRKY24 regulates terpenoid biosynthetic genes to promote saponin biosynthesis in *Conyza blinii* H. *Lév. J. Genet*. 97 1379–1388. 10.1007/s12041-018-1026-5 30555086

[B40] Suttipanta N. Pattanaik S. Kulshrestha M. Patra B. Singh S. K. Yuan L. (2011). The transcription factor CrWRKY1 positively regulates the terpenoid indole alkaloid biosynthesis in *Catharanthus roseus*. *Plant Physiol*. 157 2081–2093. 10.1104/pp.111.181834 21988879 PMC3327198

[B41] Tai Y. Liu C. Yu S. Yang H. Sun J. Guo C. (2018). Gene co-expression network analysis reveals coordinated regulation of three characteristic secondary biosynthetic pathways in tea plant (*Camellia sinensis*). *BMC Genomics* 19:616. 10.1186/s12864-018-4999-9 30111282 PMC6094456

[B42] Takehiko S. Tomoko E. Hiroshi F. Ana R. Leandro P. Mitsuo O. (2014). Characterization of three linalool synthase genes from *Citrus unshiu* marc. and analysis of linalool-mediated resistance against *Xanthomonas citri* subsp. citri and *Penicilium italicum* in citrus leaves and fruits. *Plant Sci*. 229 154–166. 25443842 10.1016/j.plantsci.2014.09.008

[B43] Tang H. Zhao T. Sheng Y. Zheng T. Fu L. Zhang Y. (2017). *Dendrobium officinale* Kimura et Migo: a review on its ethnopharmacology, phytochemistry, pharmacology, and industrialization. *Evid. Based Complement. Alternat. Med*. 2017:7436259. 10.1155/2017/7436259 28386292 PMC5366227

[B44] Teixeira da Silva J. A. Ng T. B. (2017). The medicinal and pharmaceutical importance of *Dendrobium* species. *Appl. Microbiol. Biotechnol*. 101 2227–2239. 10.1007/s00253-017-8169-9 28197691

[B45] Wagner W. H. Elmadfa I. (2003). Biological relevance of terpenoids. *Ann. Nutr. Metab*. 47 95–106. 12743459 10.1159/000070030

[B46] Yu Z. Zhao C. Zhang G. Teixeira da Silva J. A. Duan J. (2020). Genome-wide identification and expression profile of TPS gene family in *Dendrobium officinale* and the role of DoTPS10 in linalool biosynthesis. *Int. J. Mol. Sci*. 21:5419. 10.3390/ijms21155419 32751445 PMC7432446

[B47] Zhang F. Fu X. Lv Z. Lu X. Shen Q. Zhang L. (2015). A basic leucine zipper transcription factor, AabZIP1, connects abscisic acid signaling with artemisinin biosynthesis in *Artemisia annua*. *Mol. Plant* 8 163–175. 10.1016/j.molp.2014.12.004 25578280

[B48] Zhang F. Xiang L. Yu Q. Zhang H. Zhang T. Zeng J. (2018). Artemisinin biosynthesis promoting kinase 1 positively regulates artemisinin biosynthesis through phosphorylating AabZIP1. *J. Exp. Bot*. 69 1109–1123. 10.1093/jxb/erx444 29301032 PMC6019033

[B49] Zhao C. Yu Z. Silva J. He C. Wang H. Si C. (2020). Functional characterization of a *Dendrobium officinale* geraniol synthase DoGES1 involved in floral scent formation. *Int. J. Mol. Sci*. 21:7005. 10.3390/ijms21197005 32977586 PMC7582308

[B50] Zhou F. Pichersky E. (2020). The complete functional characterisation of the terpene synthase family in tomato. *New Phytol*. 226 1341–1360. 10.1111/nph.16431 31943222 PMC7422722

