# Supplementary figures and images for: Combined Analysis of Volatile Terpenoid Metabolism and Transcriptome Reveals Transcription Factors Related to Terpene Synthase in Two Cultivars of Dendrobium officinale Flowers
Source: Front Genet. 2021 Apr 22;12:661296. doi: 10.3389/fgene.2021.661296 (PMC8101708; doi:10.3389/fgene.2021.661296)

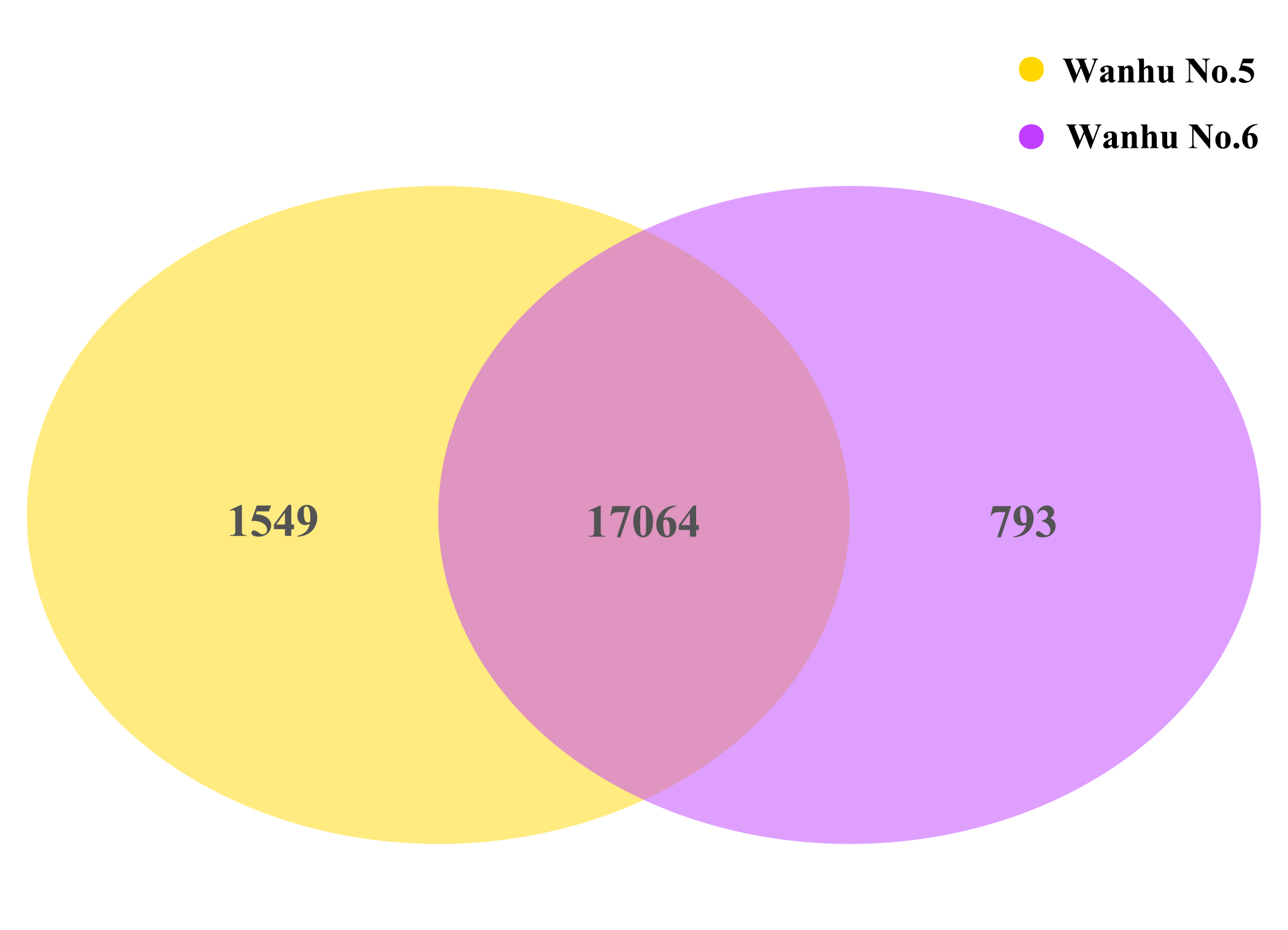

Supplement: Supplementary Figure 1 — Functional annotation of unigenes from D. officinale flower transcriptome against databases. (A) Venn diagram showing the number of unigenes annotated in two cultivars of D. officinale flowers. (B) The cluster of transcriptome gene Wanhu No.5 and Wanhu No.6. [file Image_1.TIF]

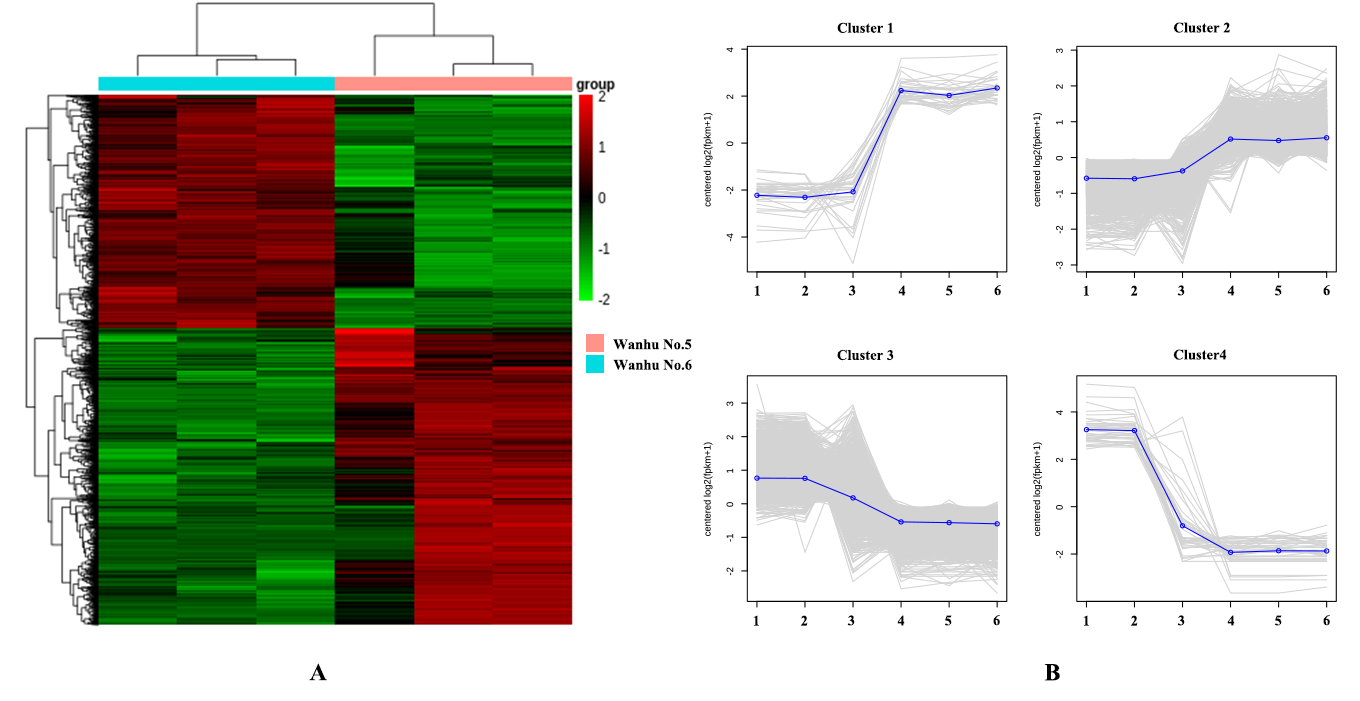

Supplement: Supplementary Figure 2 — Hierarchical cluster tree showing 10 modules of co-expressed genes. [file Image_2.TIF]

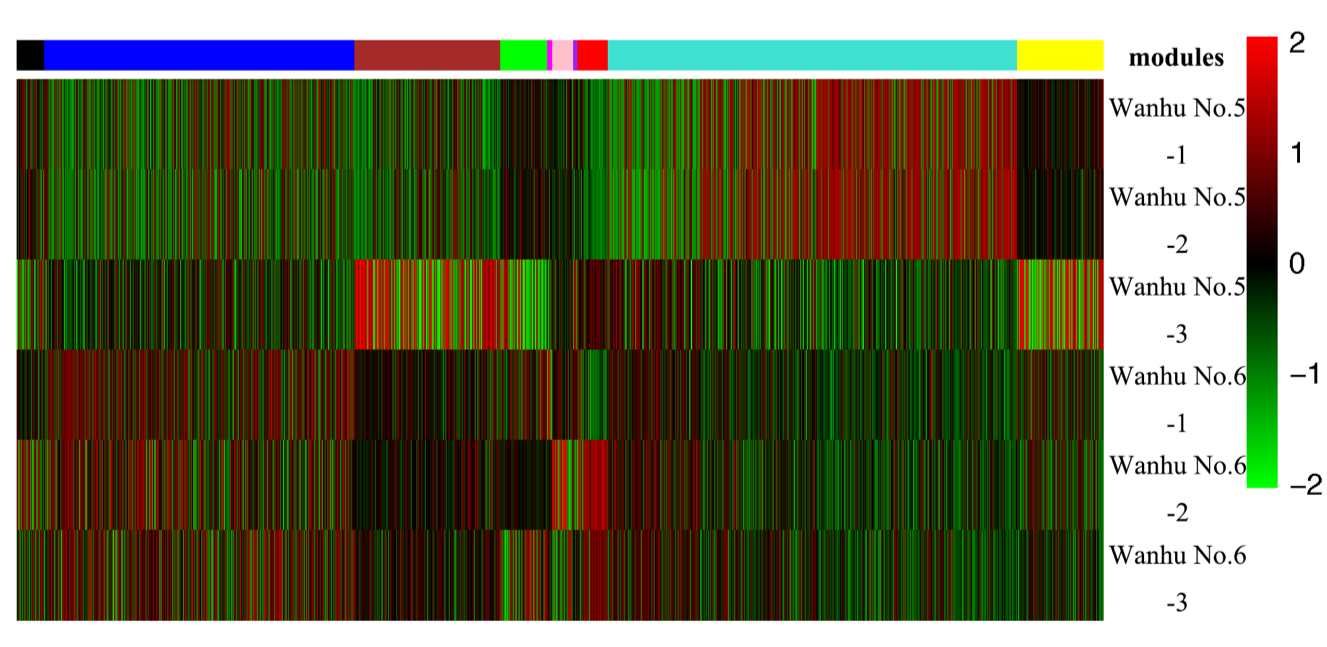

Supplement: Supplementary Figure 3 — Heatmap about DoTPS of two cultivars of D. officinale flower. [file Image_3.TIF]

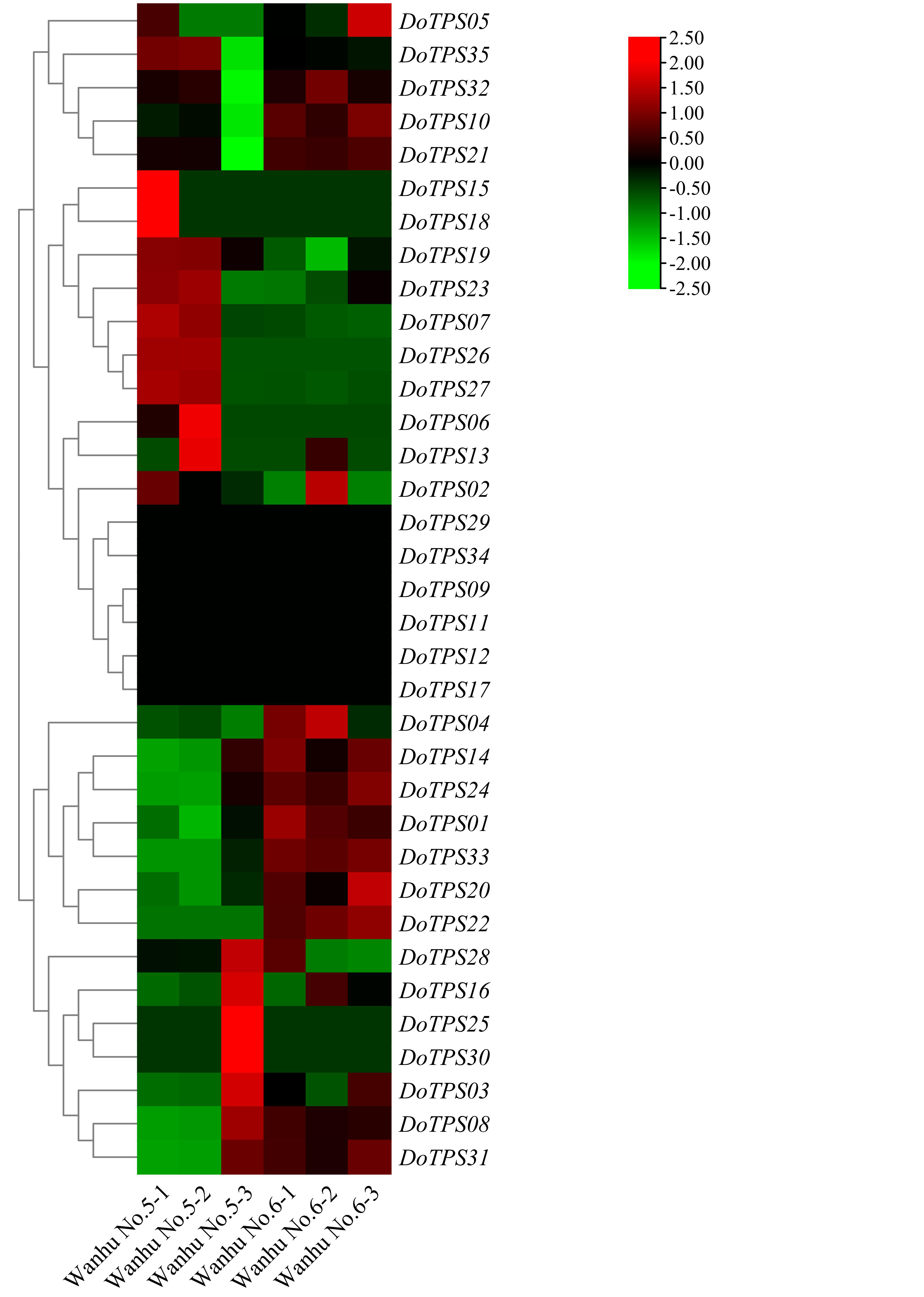

Supplement: Supplementary Figure 4 — Heatmap about DobHLH of two cultivars of D. officinale flower. [file Image_4.JPEG]

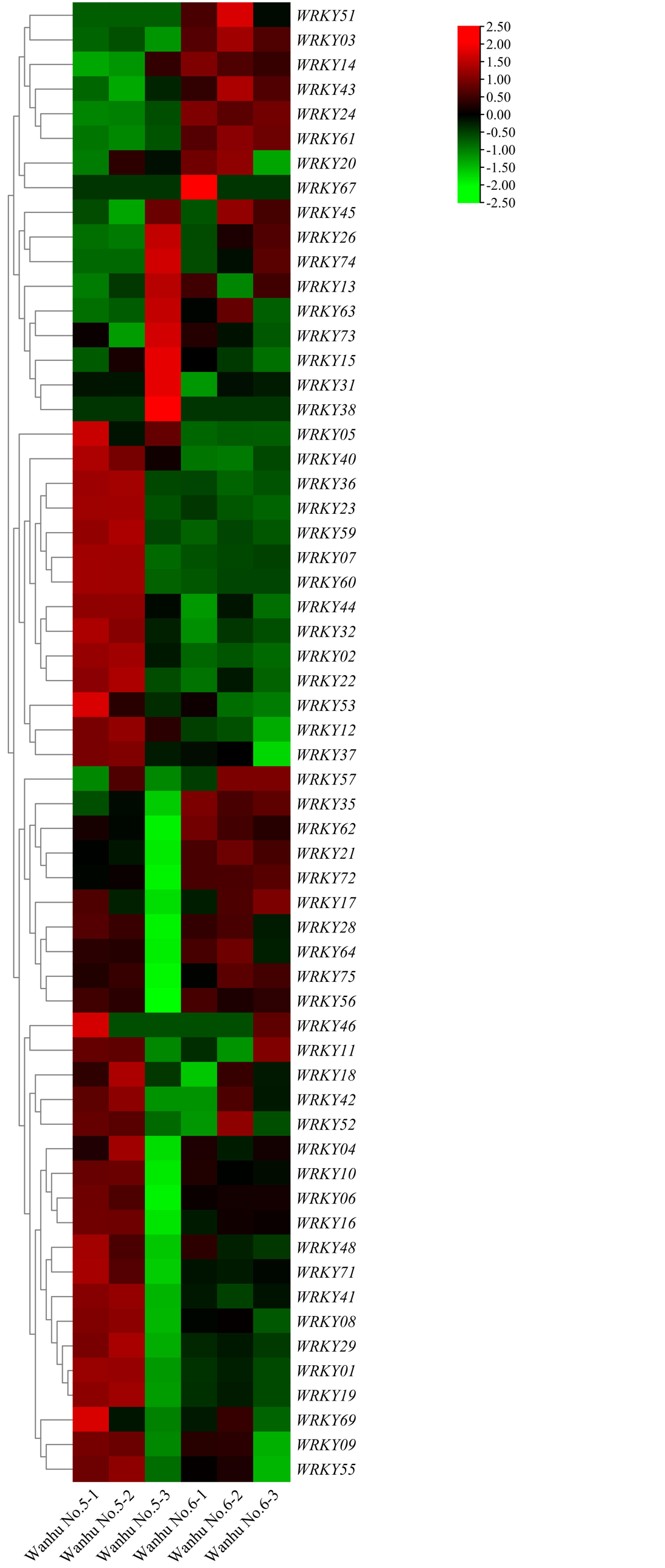

Supplement: Supplementary Figure 7 — Heatmap about DoWRKY of two cultivars of D. officinale flower. [file Image_7.TIF]

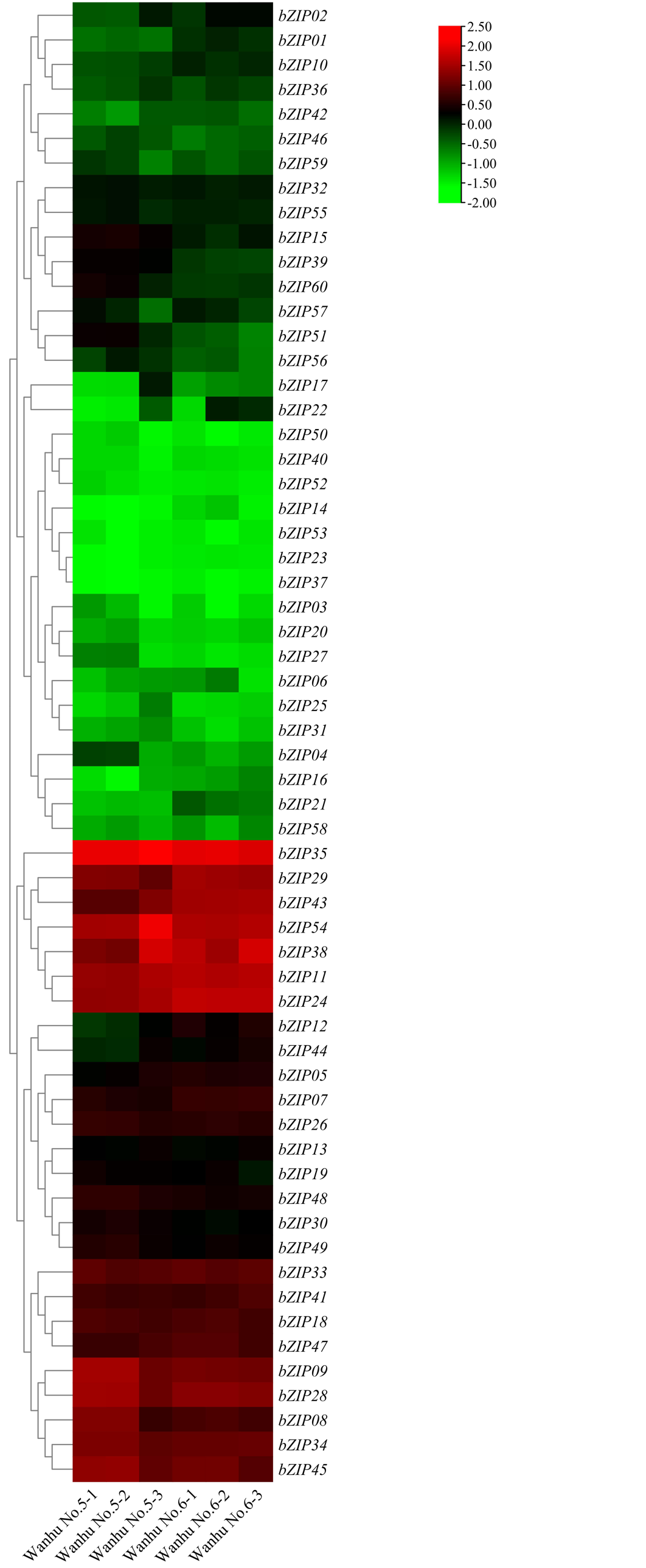

Supplement: Supplementary Figure 8 — Heatmap about DoMYB of two cultivars of D. officinale flower. [file Image_8.JPEG]
