# Supplementary material for: Combined Analysis of Volatile Terpenoid Metabolism and Transcriptome Reveals Transcription Factors Related to Terpene Synthase in Two Cultivars of Dendrobium officinale Flowers
Source: Front Genet. 2021 Apr 22;12:661296. doi: 10.3389/fgene.2021.661296 (PMC8101708; doi:10.3389/fgene.2021.661296)
Supplement: Supplementary Table 1 — Primers used for RT-qPCR analysis. [file Table_1.PDF]

Supplementary Table 1 Primers used for RT-qPCR analysis

Gene Name	Forward primer	Reverse primer
DoMVK	CGCGCTCCTGGAAAGATCA	TCATGGTTCTCTGACGGTGC
DoAACT	ACCAAGAGGAAGAGTATTGACA	TGACAATGAACCAAGGAAGGC
DoDXR	GCCATTGTTGGGTCAACTGG	TTTGATCCTGCTGCGAGAGC
DoCMK	GCCCAAGAAGAAGGAAGGCA	TCGGTTTCCCCAAGAGAGGA
DoHDS	TTCCTCCTGTCGATGATGCTG	ACAGCCTCCAGTTGTCTCCA
DoHMGS	CGGTTTGTGGACGGGAACTA	AGGCAAACAAAACAATCACAAGC
DoHMGR	ATCAGCTTTTTCGGCATCGC	GATCAGACGAAGGGGCTGG
DoPMVK	GGAATTCTTTCCTTACTATTTCAGG	ATCAAGCCCTTGCACAAGAA
UBQ	CACCAAGCCCAAGAAGATTAAGCAC	TGGTTCGCCATAAAAGTCCCAGCAC
