# Supplementary material for: Combined Analysis of Volatile Terpenoid Metabolism and Transcriptome Reveals Transcription Factors Related to Terpene Synthase in Two Cultivars of Dendrobium officinale Flowers
Source: Front Genet. 2021 Apr 22;12:661296. doi: 10.3389/fgene.2021.661296 (PMC8101708; doi:10.3389/fgene.2021.661296)
Supplement: Supplementary Table 2 — Gene IDs and relative transcript levels of DobZIP of two cultivals of D. officinale flower. [file Table_2.pdf]

Table S2 Gene IDs and relative transcript levels of *DobZIP* of two cultivals of *D. officinale* flower

Gene ID	Gene Name	Wanhu No.5-1	Wanhu No.5-2	Wanhu No.5-3	Wanhu No.6-1	Wanhu No.6-2	Wanhu No.6-3
MA16_Dca000191	bZIP01	3.611552291	4.344227113	3.363455309	7.543650627	9.851902765	7.771389525
MA16_Dca000246	bZIP02	5.107197996	5.127287798	11.02702437	7.052996526	13.79221039	12.60941097
MA16_Dca001943	bZIP03	1.912432619	1.131946501	0	0.440882043	0	0.316947056
MA16_Dca002151	bZIP04	6.935430025	6.734765497	1.291158822	1.573875001	1.166317184	1.63431359
MA16_Dca002652	bZIP05	14.66682501	16.84680277	20.23121409	21.6146607	21.01550306	20.41433253
MA16_Dca003088	bZIP06	0.889053497	1.707824284	1.777826378	1.662951955	3.106707974	0.187527008
MA16_Dca003532	bZIP07	24.18393457	21.0001067	19.39446957	26.46494662	27.7328155	26.73112261
MA16_Dca004797	bZIP08	67.66043499	67.16485419	28.04309465	31.86160048	35.97863711	28.66484267
MA16_Dca006127	bZIP09	99.82779117	98.65760075	49.72098537	55.69380598	54.95679302	50.47455919
MA16_Dca006426	bZIP10	5.537498312	5.870725054	6.806791168	9.067511029	8.363852602	8.842583354
MA16_Dca007060	bZIP11	84.95860616	82.52051241	102.2630045	115.7126951	107.5717551	112.1529047
MA16_Dca007507	bZIP12	7.716144869	9.102475872	14.14403306	20.23429353	15.6536888	20.5140042
MA16_Dca007550	bZIP13	15.00714321	14.78378029	16.68084009	12.36646915	14.11442635	15.65040216
MA16_Dca009617	bZIP14	0	0	0	0.333006649	0.846082172	0
MA16_Dca010124	bZIP15	19.65715363	20.53000331	16.01063525	9.935441214	8.47780807	10.90023145
MA16_Dca010190	bZIP16	0.405927402	0.072079105	1.275570495	1.193149385	1.783222514	2.421873468
MA16_Dca010243	bZIP17	0.397898069	0.494573548	10.80371414	1.384539184	2.447129974	2.538827186
MA16_Dca010402	bZIP18	37.89833886	36.19502218	30.94975117	33.35900186	37.15350617	29.51154959
MA16_Dca010434	bZIP19	18.47383891	16.55705807	15.63952528	14.00380593	16.74352087	10.60303083
MA16_Dca011446	bZIP20	1.37327652	1.84917986	0.471989304	0.415521571	0.527865426	0.663812418
MA16_Dca011594	bZIP21	0.864995802	1.128917882	0.891886143	4.449369778	3.590897244	2.809769218
MA16_Dca011644	bZIP22	0.138306815	0.257865887	4.658475535	0.251090575	10.63258344	8.343447633
MA16_Dca011985	bZIP23	0	0	0.110423999	0.048606561	0.246992932	0.093181122
MA16_Dca012093	bZIP24	80.38616389	81.23704702	99.68058461	130.2096908	129.2634779	122.5975094
MA16_Dca012203	bZIP25	0.498742758	0.885600018	2.889580063	0.215582817	0.492965232	0.495939195
MA16_Dca012428	bZIP26	28.16234109	27.99492356	22.56851818	22.30312033	25.01935489	21.75313294
MA16_Dca013547	bZIP27	2.903031828	3.116318021	0.326539539	0.319414541	0.243464462	0.275549889
MA16_Dca013645	bZIP28	95.1017832	92.86992185	50.2482872	67.82235423	70.41871408	61.67554933
MA16_Dca013831	bZIP29	69.66629544	66.97350133	44.79425285	92.33507163	87.57261537	78.24656338
MA16_Dca014650	bZIP30	19.6561646	21.64583563	16.11895916	13.35578668	12.99018161	14.0020166
MA16_Dca014734	bZIP31	1.252757201	1.707824284	2.222282972	0.635834571	0.372804957	0.70322628
MA16_Dca016136	bZIP32	12.02670728	12.75826699	10.41157989	10.37656078	10.03300756	10.16721016
MA16_Dca017050	bZIP33	46.12052615	38.74397696	39.61572651	43.47084198	39.36360624	40.8006739
MA16_Dca017497	bZIP34	64.07816815	63.88241666	43.02111907	44.54604331	46.20232372	45.19881734
MA16_Dca017990	bZIP35	221.0206114	219.4138321	263.8064947	196.4828749	204.1741843	168.9759492
MA16_Dca018181	bZIP36	4.889593183	5.797706085	7.783518469	4.866701653	7.616844227	6.120284443
MA16_Dca018196	bZIP37	0	0	0	0	0	0
MA16_Dca018475	bZIP38	62.30722031	56.25774112	164.0448885	122.6697026	88.0529803	156.5485205
MA16_Dca020619	bZIP39	16.64557412	16.92618787	14.12141521	6.904395301	6.739104698	5.841721028
MA16_Dca020950	bZIP40	0.490711346	0.538179921	0.039683624	0.279487723	0.399433882	0.200921794
MA16_Dca020967	bZIP41	32.40711982	30.11386831	29.93388389	26.68265929	31.62104695	34.79416777
MA16_Dca021862	bZIP42	2.991002489	2.077545347	4.837622796	4.365332062	5.139805302	3.367831982
MA16_Dca021985	bZIP43	42.0745674	41.82852222	64.14520532	90.38971262	94.08580046	95.0280954
MA16_Dca022362	bZIP44	9.593432133	9.184937326	16.75345158	12.63088378	15.94621035	18.12291086
MA16_Dca022816	bZIP45	78.41907771	81.60463547	44.90151236	51.66939708	54.42315097	36.83222774

MA16_Dca022846	bZIP46	4.986729064	7.038783669	4.880307703	2.557404006	4.028567726	4.118240178
MA16_Dca023502	bZIP47	29.43860564	30.20898872	34.40616931	37.7933164	39.12259077	29.16596526
MA16_Dca024004	bZIP48	26.14028426	26.57141398	20.67151441	18.4330233	17.71156169	17.44362576
MA16_Dca024253	bZIP49	22.75277943	24.80553682	16.52898037	13.45166859	17.14231081	14.59669685
MA16_Dca024590	bZIP50	0.471959388	0.762617971	0	0.114243157	0	0.109504822
MA16_Dca025157	bZIP51	16.84762069	17.65836542	9.139529969	4.739481959	4.620681156	2.429927429
MA16_Dca025182	bZIP52	0.595930287	0.407396414	0.122891224	0.072125864	0.274879231	0.069134381
MA16_Dca025331	bZIP53	0.281123636	0	0.096620999	0.085061481	0	0.163066964
MA16_Dca025380	bZIP54	98.79655098	100.7472813	223.6569077	103.3172951	101.1307415	106.8903946
MA16_Dca025722	bZIP55	11.68143753	12.73366311	8.696726436	9.463059082	10.38617754	8.919033458
MA16_Dca027108	bZIP56	6.613916372	11.77915626	7.871292359	3.942697046	5.008677284	2.519446826
MA16_Dca028932	bZIP57	13.02472096	10.17611761	3.581232991	10.13394335	9.726901514	5.828177518
novel.1019	bZIP58	1.417602742	1.996968712	1.03941128	1.715734624	1.017153597	2.302402243
novel.1555	bZIP59	7.92842309	7.117323705	2.701675019	4.633882046	4.011316218	4.87406442
novel.710	bZIP60	19.11998271	17.04470285	9.638136641	6.593955193	7.143946604	7.226795988
