# Supplementary material for: Combined Analysis of Volatile Terpenoid Metabolism and Transcriptome Reveals Transcription Factors Related to Terpene Synthase in Two Cultivars of Dendrobium officinale Flowers
Source: Front Genet. 2021 Apr 22;12:661296. doi: 10.3389/fgene.2021.661296 (PMC8101708; doi:10.3389/fgene.2021.661296)
Supplement: Supplementary Table 3 — Gene IDs and relative transcript levels of DoMYB of two cultivals of D. officinale flower. [file Table_3.pdf]

Table S3 Gene IDs and relative transcript levels of *DoMYB* of two cultivals of *D. officinale* flower

Gene ID	Gene Name	Wanhu No.5-1	Wanhu No.5-2	Wanhu No.5-3	Wanhu No.6-1	Wanhu No.6-2	Wanhu No.6-3
novel.1733	MYB1	5.989030959	6.976591524	1.143180013	0.742828305	0.837122639	0.792408077
MA16_Dca009980	MYB2	37.54432857	35.89502061	31.34992628	25.38269853	29.73854625	28.57924559
MA16_Dca009508	MYB3	149.5817725	152.797593	44.22885134	24.52675193	18.54931981	25.43049183
MA16_Dca000603	MYB4	69.66629544	65.08248482	54.38057155	49.63921175	52.82621873	41.47656179
MA16_Dca010564	MYB5	14.40959435	17.45138092	11.12411362	9.882685902	11.92975862	7.715396904
MA16_Dca018719	MYB6	7.849761023	8.121418267	13.59881229	10.15962391	9.34846582	9.159213869
MA16_Dca018928	MYB7	7.400986289	7.870770157	5.289400793	1.746220818	1.519976133	2.169733978
MA16_Dca017079	MYB8	6.196433466	7.606276223	5.820264926	5.869242193	5.444135879	5.134668079
MA16_Dca009965	MYB9	11.83111811	7.523831639	12.6717412	17.64935241	16.49860235	11.96980903
MA16_Dca018159	MYB10	2.387865508	2.312754003	1.108476878	3.71577923	2.05027826	3.957404487
MA16_Dca013091	MYB11	1.654053018	3.177347505	6.477351918	2.305232076	3.082624967	3.314430841
MA16_Dca015691	MYB12	1.826914263	1.380284903	0.344730877	0.693686981	0.385541599	0.24934339
MA16_Dca009507	MYB13	0.470243172	1.607364032	4.525376234	3.414831821	3.976586207	0.818299672
MA16_Dca019766	MYB14	0.310586375	0.38604813	1.164515094	0.512597571	0.173650053	0.491337139
MA16_Dca010931	MYB15	102.9160771	102.7877986	59.93814644	78.86635397	100.5508227	45.59098171
MA16_Dca000507	MYB16	46.24192987	51.82363344	58.11653124	92.34274384	76.92637456	114.8441263
MA16_Dca025723	MYB17	35.60467834	40.5013437	28.30486733	38.8193738	37.28049569	37.80149689
MA16_Dca026405	MYB18	11.20746227	11.73862823	13.14514049	4.837678414	3.795832289	3.500504151
MA16_Dca025410	MYB19	3.530836105	3.291530441	9.222853563	7.798947533	8.957497941	7.782690848
MA16_Dca017118	MYB20	9.849133882	11.35434476	7.648787903	15.92430052	14.3342061	15.43826997
MA16_Dca003815	MYB21	0.906426675	1.314433204	0.706145991	0.804506718	0.929108927	0.525776658
MA16_Dca019374	MYB22	2.085756005	3.111027159	1.43373095	2.650625507	1.924154616	2.298718164
MA16_Dca019541	MYB23	470.6695791	472.3470777	510.9894866	451.0761322	397.9956195	421.7387059
MA16_Dca020643	MYB24	39.59646204	39.75396113	49.97945704	58.54913527	56.568339	60.0931618
MA16_Dca003827	MYB25	0.051113388	0.063532175	0.843237808	1.422846592	1.650361865	1.363832786
MA16_Dca003829	MYB26	0.533265453	0.607594651	0.18328107	0	0.068326223	0
MA16_Dca019202	MYB27	29.59919522	29.68662459	22.08016464	17.89273505	17.67371648	21.74387235
MA16_Dca007146	MYB28	20.33763735	19.38444669	21.98588194	25.87902804	25.37563088	25.72664544
MA16_Dca001728	MYB29	44.86771213	46.10311024	53.09454452	77.67492596	82.89032736	78.40656257
MA16_Dca010623	MYB30	31.48016791	30.57780061	32.4438349	28.3515358	28.65284338	23.99335457
MA16_Dca009422	MYB31	32.68595428	32.14728064	42.30000691	22.39756791	27.97322711	19.39934566
MA16_Dca016418	MYB32	23.27703702	22.16470402	21.14677691	30.58870551	29.16163218	26.4248584
MA16_Dca015806	MYB33	21.12314185	19.53645029	23.3232528	21.13682076	24.99751137	25.95229077
MA16_Dca004783	MYB34	19.795107	15.29336652	20.07965714	17.82932688	21.49158241	15.53621815
MA16_Dca002275	MYB35	15.21374969	14.23824194	13.90272531	13.26841372	13.48461759	16.14419364
MA16_Dca012107	MYB36	1.244471993	0.807044702	9.149483031	1.768139908	58.23452604	40.42423204
MA16_Dca027478	MYB37	17.38357956	14.84396292	17.48681683	26.59867865	34.33336725	21.3965242
MA16_Dca003941	MYB38	2.041845353	2.537943208	13.38048274	2.965511845	60.27667514	46.74357423
MA16_Dca019037	MYB39	21.71051556	20.97414042	11.63536776	11.00681734	11.55796398	11.34309707
MA16_Dca010603	MYB40	40.27405232	41.48036212	36.70351231	47.6424117	49.91140459	49.49393175
MA16_Dca005171	MYB41	7.515648485	8.600287256	4.06735186	2.974326917	4.989074946	3.017039687
MA16_Dca000984	MYB42	10.32827825	12.1480648	8.097031026	7.696520016	9.777414931	6.089986005
MA16_Dca006844	MYB43	0.382594297	0.665772084	4.996849286	1.6670037	12.29447511	6.790918873
MA16_Dca020624	MYB44	2.166931374	1.592883275	1.345382124	1.18442365	1.218053433	0.837958523
MA16_Dca026860	MYB45	0.34950506	0	3.020245583	0	2.840418719	3.12786361

MA16_Dca012250	MYB46	0	0.109717681	0.728120018	0	0.814317995	1.075240183
MA16_Dca028065	MYB47	0.078373862	0.292248006	0	0.663995076	0.361507837	0.363688743
MA16_Dca012042	MYB48	0.253562495	0.393961773	0	0	0	0.294160013
MA16_Dca012038	MYB49	0	0.328033476	0	0	0	0
MA16_Dca017353	MYB50	1.028656939	1.232921275	0.959622192	0.88927912	0.847283993	0.383577971
MA16_Dca008055	MYB51	2.015327881	2.157068614	1.462281436	3.72650298	2.323978952	1.298888368
MA16_Dca019643	MYB52	32.18733437	33.1046448	20.67628796	27.67191304	22.75006216	22.38566918
MA16_Dca002538	MYB53	6.032273928	7.263598395	5.3386623	5.612569794	5.333031065	4.767450468
MA16_Dca019098	MYB54	5.606459339	5.798718343	3.713181675	3.615651309	2.89435072	3.275788243
MA16_Dca020353	MYB55	0.124343146	0.051518078	0	0.100328926	0.382364058	0.048083848
MA16_Dca025324	MYB56	0.155803461	0	0	0	0	0
MA16_Dca012364	MYB57	1.066530906	0.662830529	2.871403428	2.097598584	0.683262235	1.08263015
MA16_Dca010747	MYB58	429.85031	425.855792	613.9497639	455.4718267	473.7976225	516.2052975
MA16_Dca018028	MYB59	16.20120816	14.6179144	17.81180767	11.78278055	12.98017762	14.01145193
MA16_Dca002414	MYB60	56.55941787	57.88409911	27.80972133	35.74140199	38.94352519	34.85730975
MA16_Dca002932	MYB61	0.2402171	0.149290777	0	0	0	0.046446318
MA16_Dca003340	MYB62	18.8987372	19.58687885	11.51777517	21.3864006	21.99904298	16.36307093
MA16_Dca004678	MYB63	53.7793977	52.66030733	40.24095896	34.48257424	46.2041445	23.0509389
MA16_Dca006396	MYB64	98.21092197	101.5583761	93.16237324	103.9660759	104.6092948	99.51883745
MA16_Dca007528	MYB65	2.048584116	1.551663298	1.584201723	0.30992698	0.246075879	0.37134061
MA16_Dca007572	MYB66	0.243994099	0.606552465	1.006316817	0	0	0
MA16_Dca007754	MYB67	0.743858115	0.995712232	0.471989304	0.969550333	0.175955142	0.796574902
MA16_Dca010048	MYB68	9.096017292	8.899529669	12.35438669	6.631912083	7.863306059	6.95015352
MA16_Dca010990	MYB69	33.91732003	31.7242901	30.17626351	37.75535914	43.60291894	35.59723206
MA16_Dca012494	MYB70	0.164385855	0	0.677984636	0.066319121	0.252749123	0.190705432
MA16_Dca012585	MYB71	2.351215861	3.312144066	1.562332271	0.663995076	1.024272205	0.727377486
MA16_Dca016860	MYB72	23.25938284	24.0007428	19.96262383	18.53585475	19.17040279	16.66622284
MA16_Dca019092	MYB73	2.128672796	1.918253371	0.658454214	3.542478141	3.35473322	2.222542318
MA16_Dca019246	MYB74	0.138060718	0.057201567	0.25307137	0.111397242	0	0
MA16_Dca020096	MYB75	30.40820658	31.04076192	56.16900729	42.0770793	44.3763968	46.20230634
MA16_Dca020882	MYB76	2.490939754	3.227903179	0.655755631	1.988486426	3.340984313	1.475622359
MA16_Dca022978	MYB77	26.49883821	26.90458837	13.84695238	18.5643025	18.09981806	18.18136545
MA16_Dca023171	MYB78	0.601473825	0.249203726	0.551263993	0.121328004	0	0.232591638
MA16_Dca024405	MYB79	0.140561818	0	0	0	0.216118816	0.326133927
MA16_Dca024815	MYB80	0.475841305	0.313122863	0.03848109	0.169386499	0.086073295	0.194833255
MA16_Dca026491	MYB81	8.271619762	9.412492079	2.802879424	3.595571794	5.821578907	2.703092009
MA16_Dca004468	MYB82	0	0	0	0	0.064554971	0.194833255
MA16_Dca004957	MYB83	0.371600208	0.184754486	1.430435016	0.809550647	0.571348593	0.258657942
MA16_Dca008884	MYB84	84.55608752	86.00729641	72.09527962	107.9162321	92.93347876	131.0409723
MA16_Dca025622	MYB85	0	0.17663341	1.367558752	0	0.218493748	0.659435633
MA16_Dca018701	MYB86	1.841902039	1.621672911	0.896826363	1.532621107	1.887993451	2.225839859
MA16_Dca017307	MYB87	1.853688955	2.084635946	0.060676668	0.267087244	0.814317995	0.256009567
MA16_Dca017917	MYB88	0.760687484	0.746453885	0	0.339192531	0	0.139338953
MA16_Dca019356	MYB89	0.080321039	0.249590688	0.055211999	0.291639364	0.370489398	0.232952805
MA16_Dca011059	MYB90	0.193010257	0.047981016	0.106138888	0	0.178056099	0.179130276
MA16_Dca000498	MYB91	0.076745918	0.047696262	0	0.092886128	0.235999181	0.178067189
MA16_Dca019358	MYB92	0.072649928	0	0	0.043964361	0	0.168563603
MA16_Dca014204	MYB93	0.04028563	0.100147292	0.055383999	0.097515966	0	0

MA16_Dca014239	MYB94	535.5839801	559.5857873	313.4470544	215.8103691	243.0484472	195.1700647
MA16_Dca010086	MYB95	10.87631973	14.15757724	30.25836947	34.30850624	30.02190911	27.42116781
MA16_Dca011999	MYB96	5.779525021	6.914359244	4.866675559	5.421125005	7.497753323	3.981020284
MA16_Dca008519	MYB97	1.795050069	1.639238106	5.590332235	2.615941752	3.210558269	2.422445204
MA16_Dca004949	MYB98	2.954821008	2.290061658	2.198387456	1.262202622	1.924154616	0.725910999
MA16_Dca014745	MYB99	4.14413143	2.420355106	2.333826133	1.631604007	1.612129543	1.274314804
MA16_Dca019744	MYB100	1.527364634	2.341435795	2.729733414	2.403154284	2.426656937	1.122208867
MA16_Dca008230	MYB101	0.790928883	0.73732295	1.794136711	0.813676797	0.912061057	1.697492183
MA16_Dca018562	MYB102	0.741982054	0.329377875	1.020064315	0.384868341	2.118672979	0.553358385
MA16_Dca022272	MYB103	0.688983336	0.592880176	0.655755631	0.641447234	0.7333868	0.553358385
MA16_Dca022000	MYB104	0.273976424	0.953521036	0.376658131	0.265276483	0.252749123	0.190705432
MA16_Dca004823	MYB105	0	0	0.704626308	0.143152249	0.181856077	0.045738295
MA16_Dca025001	MYB106	0.05321682	0.066146668	0	0.128817387	0.327291046	0.123474573
MA16_Dca010164	MYB107	0.123748203	0.30762948	0.085063463	0	0.285400924	0
MA16_Dca018923	MYB108	0	0.075110469	0.166151998	0.365684872	0	0.070103554
MA16_Dca002764	MYB109	0	0	0.190141859	0	0.212651669	0.080225458
MA16_Dca010782	MYB110	0	0	0	0	0	0.133352539
MA16_Dca016337	MYB111	0.788517514	1.078110021	1.084040474	1.049783156	0.909280383	0.960504188
MA16_Dca004380	MYB112	0.325618024	0.40473195	0.319752946	0.337798112	0.143042669	0.16189382
MA16_Dca010713	MYB113	2.665309583	2.576690433	0.271423874	1.135018846	0.227667149	0.629861707
MA16_Dca017137	MYB114	0	0	0.194652523	0	0	0
MA16_Dca018542	MYB115	6.604150432	6.274736595	7.082784231	5.607689511	5.90108381	4.973978396
MA16_Dca015427	MYB116	1.198546622	1.136916023	2.124719329	1.374261587	1.260868797	1.28067225
MA16_Dca028079	MYB117	0.56773261	0.392040008	3.33884466	1.145217989	1.260868797	1.097719072
MA16_Dca004802	MYB118	0.213746896	0	0.881566799	0.323374226	0.246482616	0.681916393
MA16_Dca025236	MYB119	0.243229227	0.201550349	0	0	0.24931575	0.047028717
MA16_Dca020102	MYB120	0.374228891	0.206734924	0	0	0	0
MA16_Dca024576	MYB121	0	0	0.108404047	0	0.060618692	0
MA16_Dca009771	MYB122	0.686171159	0.656066952	1.233593813	1.980370155	1.055012667	1.163432867
MA16_Dca018323	MYB123	1.077640603	0.8667159	0.784335167	1.304276043	0.487326741	1.250180054
MA16_Dca011590	MYB124	0.093707879	0.116475654	0.386483995	0.170122962	0.360198026	0.163066964
MA16_Dca000003	MYB125	129.8652124	129.4978796	102.5234151	92.41464033	104.965014	86.35519327
MA16_Dca017755	MYB126	60.88438153	57.22353925	40.9739384	69.32255157	65.19553352	77.55408798
MA16_Dca000602	MYB127	37.25097964	39.29645201	43.25159695	31.53622671	39.40974987	31.84040663
MA16_Dca012586	MYB128	41.73932978	45.35062804	56.43011404	45.55649893	55.83198045	49.62768321
MA16_Dca012989	MYB129	26.90421759	29.01096058	16.99775463	19.16331435	21.58025442	21.44211254
MA16_Dca020947	MYB130	21.39671927	22.43145905	13.56892556	11.77118211	11.57529968	11.45011704
MA16_Dca021769	MYB131	10.14249979	6.776142488	25.97020885	14.42375859	7.60229716	12.35472053
MA16_Dca017188	MYB132	0.25949874	0.483821949	0.059459076	0.052345527	0.332490486	0.301046702
MA16_Dca011133	MYB133	0.15154321	0.125575315	0.069446343	0.550241456	0.155335399	0.17580657
MA16_Dca013023	MYB134	0	0	0.304422325	0.268001927	0.06809223	0.154131787
MA16_Dca019829	MYB135	1.086696406	0.945508254	0.224095762	1.578283615	2.172084903	1.449788634
MA16_Dca015542	MYB136	0.77407987	0.396181275	0.876393285	0.551102553	0.560082564	0.898016658
MA16_Dca008861	MYB137	0.607899827	1.236433871	0.455852917	0.267543804	0.339879163	0.192335393
MA16_Dca009488	MYB138	0.203328416	0.606552465	0.614971388	0.492179639	0.125049881	0.188706423
MA16_Dca025130	MYB139	0.331581724	0.463662702	0.284908073	0.15049339	0.127454686	0.144251545
MA16_Dca001932	MYB140	0	0	0	0	0	0.097733946
MA16_Dca012056	MYB141	11.26721264	10.50356694	76.74577234	39.98058048	11.41792079	64.7618024

MA16_Dca016309	MYB142	0.276121436	0.514814103	1.581696065	1.448164147	0.84909314	1.441488745
MA16_Dca019344	MYB143	11.40822819	12.90294963	11.27580292	12.32808862	12.93752362	13.37863915
MA16_Dca012068	MYB144	32.81415636	33.61113003	26.03245767	17.30029008	18.64024785	19.79749414
MA16_Dca002190	MYB145	44.69677131	44.76886398	38.59911632	37.5526458	37.23045476	33.27660466
MA16_Dca018512	MYB146	22.97529765	23.89614528	30.87491809	23.94650815	21.27473621	23.65340662
MA16_Dca012703	MYB147	4.149556685	4.502802466	2.203418091	2.896422859	1.822883321	2.725350067
MA16_Dca023703	MYB148	35.99769482	36.41731792	25.26739956	26.52110451	22.71257018	30.31745937
MA16_Dca024789	MYB149	0.758827613	0.314398833	0.738949839	0.459207213	0.631975803	0.513521391
