# Supplementary material for: Combined Analysis of Volatile Terpenoid Metabolism and Transcriptome Reveals Transcription Factors Related to Terpene Synthase in Two Cultivars of Dendrobium officinale Flowers
Source: Front Genet. 2021 Apr 22;12:661296. doi: 10.3389/fgene.2021.661296 (PMC8101708; doi:10.3389/fgene.2021.661296)
Supplement: Supplementary Table 4 — Gene IDs and relative transcript levels of DoAP2/ERF of two cultivals of D. officinale flower. [file Table_4.pdf]

Table S4 Gene IDs and relative transcript levels of *DoAP2/ERF* of two cultivals of *D. officinale* flower

Gene ID	Gene Name	Wanhu No.5-1	Wanhu No.5-2	Wanhu No.5-3	Wanhu No.6-1	Wanhu No.6-2	Wanhu No.6-3
MA16_Dca000204	AP2/ERF01	0.813882413	0.899224633	2.859441027	1.641746068	0.556165903	2.203114501
MA16_Dca000285	AP2/ERF02	0	0	0	0	0	0
MA16_Dca000925	AP2/ERF03	0.157224161	0.146568149	1.242857346	0.285434271	2.236074311	0.820786905
MA16_Dca001584	AP2/ERF04	0.11443971	0	0.681762328	0.092338127	0.234606856	0.75232074
MA16_Dca001811	AP2/ERF05	0.34950506	0.108605678	0.240246808	0.846016893	0.671720643	0.405463801
MA16_Dca001859	AP2/ERF06	24.8799762	26.95237787	8.382147179	11.12849977	13.83489118	7.928898593
MA16_Dca001938	AP2/ERF07	0.608073067	0.554263459	2.452174314	1.22659189	0.186986812	0.987603052
MA16_Dca002039	AP2/ERF08	23.67698611	25.71782451	24.22975743	35.56236369	33.98546356	45.93445064
MA16_Dca002334	AP2/ERF09	2.570518869	2.506898032	0.761148908	1.579490254	0.972865127	1.192832345
MA16_Dca002515	AP2/ERF10	0	0.132840003	0.587711199	0	1.478895697	0.743908792
MA16_Dca002518	AP2/ERF11	0	0	3.07891719	0.0874375	1.11077827	1.2571643
MA16_Dca003321	AP2/ERF12	61.92752085	61.09980047	54.77030331	54.43934788	46.43467124	44.8201197
MA16_Dca003385	AP2/ERF13	10.00226421	9.11933063	1.451286839	1.437365435	1.825983462	2.173782461
MA16_Dca003422	AP2/ERF14	0	0.073062001	0	0	0.180753919	0.068191639
MA16_Dca003553	AP2/ERF15	108.8277056	119.5607467	51.25681244	106.8151263	96.44512651	111.0549554
MA16_Dca003726	AP2/ERF16	0.795796137	1.137519161	1.066695827	1.179868174	0.825906366	0.877049392
MA16_Dca003949	AP2/ERF17	19.90608035	25.94658793	14.58217141	10.19973175	15.56379246	10.50713124
MA16_Dca003950	AP2/ERF18	6.979005809	5.613017254	9.947361874	8.695173619	6.627643678	8.334533693
MA16_Dca004012	AP2/ERF19	8.575750481	10.21945132	3.630508603	3.196162766	4.60446824	3.095182618
MA16_Dca004231	AP2/ERF20	0.087970661	0.218688984	0	0.319414541	1.082064274	0.714388602
MA16_Dca004232	AP2/ERF21	69.33799614	76.67814409	53.74984807	93.48157475	101.7356199	95.20826465
MA16_Dca004258	AP2/ERF22	23.5937314	21.51899602	11.02977997	18.78157502	10.71243631	16.59422668
MA16_Dca004554	AP2/ERF23	0	0	0	0	0	0
MA16_Dca004576	AP2/ERF24	6.984998944	6.218269613	16.86988533	12.62386885	12.04586333	13.63335037
MA16_Dca004577	AP2/ERF25	3.870066982	3.226460648	2.141177754	2.856078926	3.845968412	2.190096445
MA16_Dca004580	AP2/ERF26	7.243891189	6.936342711	12.39314653	9.936310435	10.97273787	11.20493326
MA16_Dca004865	AP2/ERF27	17.29151322	17.77285372	10.61616323	31.03431682	33.00566552	24.73213341
MA16_Dca005005	AP2/ERF28	109.1913988	120.1265106	91.18718738	76.18354105	108.5660705	66.9632866
MA16_Dca005355	AP2/ERF29	0.598689224	0.347270007	0.823067767	0.772904322	0.613670711	0.601938544
MA16_Dca005447	AP2/ERF30	3.51441632	4.368298029	0.703625215	1.321482851	1.993539259	1.029171971
MA16_Dca005472	AP2/ERF31	0	0	0	0.120859556	0.383840367	0.1158468
MA16_Dca005593	AP2/ERF32	0	0	0	0	0	0
MA16_Dca005811	AP2/ERF33	130.9589914	126.5905482	204.403001	152.7866222	172.371336	127.7961833
MA16_Dca005958	AP2/ERF34	0.508549498	0.632109451	0.599267318	0.615501054	0.893614878	0.168563603
MA16_Dca006007	AP2/ERF35	0.226871706	0.140996845	0.623798729	0.068646108	0.348823351	0.06579895
MA16_Dca006176	AP2/ERF36	0.075623902	0	0.727765184	0.27458443	0	0.350927734
MA16_Dca006212	AP2/ERF37	62.85521285	65.83148306	22.09887768	24.19406078	38.65685396	18.58833451
MA16_Dca006263	AP2/ERF38	2.125756806	3.082615952	1.2176893	0.536003853	0.408553377	0.513772625
MA16_Dca006394	AP2/ERF39	18.79135801	18.33399599	17.13009791	29.75379723	16.87977999	28.91041375
MA16_Dca006431	AP2/ERF40	0.753302169	1.248438083	0.086302251	0.075977245	0.579114496	0.145652045
MA16_Dca006653	AP2/ERF41	18.91721104	21.95200021	7.212895589	26.02589681	31.69907291	20.31721185
MA16_Dca007398	AP2/ERF42	4.103782665	3.380802576	4.198556759	3.754004847	4.402124952	4.650116215
MA16_Dca007407	AP2/ERF43	30.31097788	35.63145123	62.8405104	45.4159473	39.19121088	59.44497759
MA16_Dca007470	AP2/ERF44	56.38460784	61.7806744	55.01993481	59.93488394	45.98516751	61.78614978
MA16_Dca008681	AP2/ERF45	1.296493065	0.661126594	0.137107433	1.609389462	1.533387998	1.388374764

MA16_Dca008693	AP2/ERF46	0	0	0	0	0	0
MA16_Dca009059	AP2/ERF47	8.588956446	6.417460874	8.424774252	7.475253738	7.5673842	5.541842926
MA16_Dca009728	AP2/ERF48	82.54339178	86.1530593	55.20859291	87.83242987	100.9480432	79.52302122
MA16_Dca009859	AP2/ERF49	1.873281796	1.201767501	0.415379995	0.511958821	0.278732678	0.140207109
MA16_Dca011128	AP2/ERF50	0.141219673	0.225682937	0	0	0	0
MA16_Dca011474	AP2/ERF51	0.082367435	0	0	0	0	0
MA16_Dca011493	AP2/ERF52	14.74454058	14.42121001	29.74120762	20.99031164	24.99303013	23.48469073
MA16_Dca012028	AP2/ERF53	19.27810894	18.44863731	18.05971386	19.73965008	19.20093076	20.74177356
MA16_Dca012097	AP2/ERF54	0	0	0	0.094285015	0	0
MA16_Dca012098	AP2/ERF55	0.635984618	0.790506901	1.360085753	0.513157787	3.042197098	1.393643339
MA16_Dca012187	AP2/ERF56	0	0	1.143909734	0.587448727	0.746276232	0.241321619
MA16_Dca012503	AP2/ERF57	117.0757121	110.2526882	99.24758904	90.86878528	125.9574029	86.00753264
MA16_Dca012658	AP2/ERF58	2.469712151	2.686045995	0.84882957	0.871823774	0.685618312	0.437728825
MA16_Dca012727	AP2/ERF59	0	0	0.194297965	0	0	0
MA16_Dca012732	AP2/ERF60	0.136843251	0.170091432	0	0	0.210401387	0
MA16_Dca012809	AP2/ERF61	0.232305759	0.144374015	6.227715155	0.468602171	7.20309777	6.692580648
MA16_Dca012817	AP2/ERF62	0	0.193658317	0	0	0	0.060249641
MA16_Dca012912	AP2/ERF63	0.048983664	0.121770002	0	0.948564395	0.979083725	0.738742759
MA16_Dca013018	AP2/ERF64	1.006658288	1.539989492	9.3681869	0.65604304	3.214605616	1.886499243
MA16_Dca013083	AP2/ERF65	0.769170988	0.657286407	0.594811799	2.21096608	0.443485451	1.115402279
MA16_Dca013337	AP2/ERF66	5.580479138	4.035083981	3.688436468	2.760086232	3.341322435	3.921726642
MA16_Dca013671	AP2/ERF67	92.33224685	95.07558249	95.91373307	76.14363538	85.49660345	74.93579243
MA16_Dca014041	AP2/ERF68	736.4015612	769.8861569	276.1898863	257.2935317	275.5570314	263.6437398
MA16_Dca014263	AP2/ERF69	0	0	0	0	0	0
MA16_Dca014264	AP2/ERF70	0	0	0	0	0	0
MA16_Dca014326	AP2/ERF71	0.813313663	1.718565317	1.956727145	0.196871856	2.250897853	1.179415145
MA16_Dca014404	AP2/ERF72	101.1474977	111.447653	34.68626289	34.31249282	41.2953183	24.65390036
MA16_Dca014713	AP2/ERF73	41.05297535	43.37331515	13.07501939	13.58103308	20.19853312	13.65275995
MA16_Dca014976	AP2/ERF74	9.28730265	7.403616147	6.410949664	4.932534853	6.205884535	4.046037266
MA16_Dca015060	AP2/ERF75	10.71686787	13.40950104	8.152463499	6.917707189	5.053120595	5.387516255
MA16_Dca015347	AP2/ERF76	0.147790711	0.27554812	0.304770236	0.178872143	0.113616749	0
MA16_Dca016047	AP2/ERF77	37.63452567	36.16569072	88.77735565	143.9720094	120.6995878	140.5010045
MA16_Dca016049	AP2/ERF78	24.16741549	20.65199279	27.28672043	20.10937079	19.43475021	19.2138328
MA16_Dca016529	AP2/ERF79	0.284838926	0.495662917	0.078318343	0.068948513	0.613130032	0.066088813
MA16_Dca016530	AP2/ERF80	0.186067442	0.1156377	0	0.112599371	0.429128008	0.32378764
MA16_Dca016545	AP2/ERF81	12.02837085	14.35990661	12.28791761	8.573696929	11.03795068	8.273250357
MA16_Dca016546	AP2/ERF82	2.855047831	3.131228634	5.26421317	3.008304224	3.61507837	2.64973227
MA16_Dca016670	AP2/ERF83	0.627751807	0.468164281	0.086302251	0.075977245	0	0.072826023
MA16_Dca016893	AP2/ERF84	0.177755151	0.773302283	0.610936899	0.537845791	1.161545799	1.031076333
MA16_Dca017057	AP2/ERF85	432.8115732	450.3933442	319.0923426	303.2643492	295.1692855	273.4414436
MA16_Dca017224	AP2/ERF86	37.25557513	41.71492369	19.40088309	14.65757839	14.75439724	12.73993007
MA16_Dca017496	AP2/ERF87	22.63045266	23.41763116	86.49023153	45.73961157	36.45204023	64.75070486
MA16_Dca018207	AP2/ERF88	0.087376265	0.108605678	0.840863827	0	1.880817801	1.115025454
MA16_Dca018529	AP2/ERF89	0.195934655	0.208748576	0.192405452	0.101631899	0.172146589	0
MA16_Dca018942	AP2/ERF90	16.79439901	30.05979488	19.39446957	5.894650168	6.713716973	0.974166276
MA16_Dca019542	AP2/ERF91	14.32970748	14.66176651	8.408638272	11.26259988	10.00863758	8.210641976
MA16_Dca020119	AP2/ERF92	74.52442169	74.61771821	45.59511615	53.09302995	58.02044962	41.25594178
MA16_Dca020229	AP2/ERF93	1.538341976	1.613339363	1.850525597	0.349099907	0.813056659	0.836551709

MA16_Dca020230	AP2/ERF94	7.090131966	6.928293241	6.620870647	5.343034272	7.198992271	6.466448555
MA16_Dca020428	AP2/ERF95	164.8134167	171.731705	308.5172905	233.1365073	270.4654938	210.9434241
MA16_Dca020530	AP2/ERF96	4.750415719	3.389679251	1.814108549	2.02295876	1.623096411	1.020555146
MA16_Dca020701	AP2/ERF97	2.368675384	1.114014676	1.232156895	3.33171504	3.248201605	1.633898684
MA16_Dca020702	AP2/ERF98	7.0420079	5.331355948	3.080392238	5.888612629	5.9058211	3.044993003
MA16_Dca021371	AP2/ERF99	0.69632162	0.370930161	2.666739566	2.949670435	0.841200928	2.423425951
MA16_Dca021421	AP2/ERF100	3.045506051	3.144842671	0.193241998	4.253074053	6.411524863	4.131029743
MA16_Dca021484	AP2/ERF101	0.333004821	0.965798131	0.076301561	0.470211106	0.341337872	0.772643467
MA16_Dca021629	AP2/ERF102	21.82222221	20.71992697	7.152973316	7.947932136	11.33948411	6.915058423
MA16_Dca021721	AP2/ERF103	76.59831258	73.96719439	6.345633973	22.80751735	32.72765639	14.86939816
MA16_Dca021730	AP2/ERF104	0	0	0	0	0	0
MA16_Dca022261	AP2/ERF105	21.18457306	20.27378754	6.879026687	15.33671327	12.08962138	9.197304517
MA16_Dca022373	AP2/ERF106	0.350557786	0.629389531	1.445822656	0	0.059888346	0.045187231
MA16_Dca022576	AP2/ERF107	2.732985446	3.065591195	1.191326954	2.258952322	3.382148063	2.62924465
MA16_Dca022999	AP2/ERF108	0	0	0	0	0	0
MA16_Dca023270	AP2/ERF109	0.555008036	0.413913485	0.991920296	1.343460302	1.024013616	0.708256511
MA16_Dca023272	AP2/ERF110	1.626140311	0.866244089	1.064566693	1.030924776	2.143070411	0.898332973
MA16_Dca023523	AP2/ERF111	56.34309495	58.40947558	64.40043834	75.57041054	72.29743116	85.65306823
MA16_Dca024190	AP2/ERF112	0.07790173	0.484145793	0	0	0.119776693	0
MA16_Dca024506	AP2/ERF113	5.02201446	4.291505911	0.086302251	2.507249092	1.351267158	1.966302609
MA16_Dca024926	AP2/ERF114	18.31989025	16.00666682	50.29767126	35.67194977	35.78927586	40.56834275
MA16_Dca024988	AP2/ERF115	8.621124823	8.602793411	12.68683612	6.907152002	6.814337867	8.487137831
MA16_Dca025176	AP2/ERF116	0	0	0	0	0	0
MA16_Dca025483	AP2/ERF117	2.604298124	2.00920504	4.197645614	4.130207469	3.175745929	3.750540162
MA16_Dca025501	AP2/ERF118	103.4147802	102.6017999	36.94046425	30.69344221	31.07452096	32.74423686
MA16_Dca025668	AP2/ERF119	0.61579463	1.148117166	0.507950394	0.894360715	0.662764368	0.714388602
MA16_Dca025795	AP2/ERF120	0.047542968	0	0.261445056	0	0	0.055155002
MA16_Dca026159	AP2/ERF121	0	0	0.026855383	0.070927398	0	0
MA16_Dca026998	AP2/ERF122	0.071053227	0.264950115	0	0.085996223	0.218493748	0.082429454
MA16_Dca027453	AP2/ERF123	1.436854137	0.85045716	0	0.662489419	0.841605546	0.476259068
MA16_Dca028380	AP2/ERF124	29.12024385	34.29043268	42.53614222	33.62133799	32.54909451	24.22571127
MA16_Dca028782	AP2/ERF125	0.248686293	0.206072312	0.113963229	0.100328926	0.764728117	0.192335393
