# Supplementary material for: Combined Analysis of Volatile Terpenoid Metabolism and Transcriptome Reveals Transcription Factors Related to Terpene Synthase in Two Cultivars of Dendrobium officinale Flowers
Source: Front Genet. 2021 Apr 22;12:661296. doi: 10.3389/fgene.2021.661296 (PMC8101708; doi:10.3389/fgene.2021.661296)
Supplement: Supplementary Table 5 — Gene IDs and relative transcript levels of DoWRKY of two cultivals of D. officinale flower. [file Table_5.pdf]

Table S5 Gene IDs and relative transcript levels of *DoWRKY* of two cultivals of *D. officinale* flower

Gene ID	Gene Name	Wanhu No.5-1	Wanhu No.5-2	Wanhu No.5-3	Wanhu No.6-1	Wanhu No.6-2	Wanhu No.6-3
MA16_Dca000627	WRKY62	3.707083674	3.53620087	2.37043517	4.278025421	3.976586207	3.800547364
MA16_Dca000637	WRKY46	0.137571141	0	0	0	0	0.079798727
MA16_Dca000671	WRKY06	18.62353484	17.61292079	10.14965335	15.70896007	15.96493173	15.99677903
MA16_Dca000873	WRKY41	41.05112944	43.47977525	9.752464841	20.21158702	16.66447098	20.56051513
MA16_Dca002096	WRKY01	63.05648569	62.04367448	2.904527834	9.019876692	10.95881477	7.056671615
MA16_Dca002205	WRKY26	0.048433286	0	3.196092364	0.234476592	0.968082784	1.517072425
MA16_Dca002550	WRKY14	0.097475029	0.121158093	0.446690045	0.589873587	0.499571132	0.452326452
MA16_Dca002715	WRKY28	2.860279731	2.553755939	0.387687995	2.486657129	2.725386186	1.729221009
MA16_Dca003067	WRKY57	0	0.213366907	0	0.069253595	0.263932713	0.265524967
MA16_Dca003180	WRKY11	8.288559177	8.203757077	5.433712667	6.40914281	5.309981582	8.680775449
MA16_Dca004998	WRKY31	0.87104629	0.860592141	3.254742591	0.216253023	0.89284319	0.829134958
MA16_Dca005043	WRKY63	5.085002438	5.448691634	14.52393752	7.427741532	10.64916306	5.390606877
MA16_Dca005648	WRKY59	9.459755642	10.19456565	4.703976407	4.263003409	4.719294915	4.436436612
MA16_Dca005780	WRKY09	14.57066152	14.34986552	10.52334555	13.18808367	13.26451806	10.00840522
MA16_Dca006278	WRKY10	159.1142842	161.1721344	44.33856146	122.2876646	106.9605868	102.8913247
MA16_Dca006505	WRKY20	34.99162428	39.10727195	37.58998909	40.86731601	41.68268059	34.09804814
MA16_Dca006787	WRKY17	1.585629225	1.051137242	0.387537085	1.066165703	1.57113079	1.839501987
MA16_Dca007186	WRKY02	60.93475784	66.73453485	13.02070023	6.172348597	7.405536129	6.104165522
MA16_Dca007842	WRKY69	0.317064135	0.112599932	0.031135313	0.109641419	0.174106226	0.052546972
MA16_Dca008357	WRKY36	11.8220375	12.51940302	0.704089654	0.723162954	0.328101172	0.544632895
MA16_Dca008849	WRKY07	17.72120102	17.65253253	13.28359805	13.60983697	13.76756899	13.86673142
MA16_Dca008968	WRKY75	4.467310135	5.114340102	0.32324116	3.557116481	6.326387148	5.455331145
MA16_Dca008985	WRKY24	25.96479947	26.22209558	29.14240886	45.08191781	41.75415517	43.9475059
MA16_Dca009368	WRKY13	1.327587663	1.650145584	3.177104173	2.320917065	1.28520847	2.338739873
MA16_Dca010430	WRKY29	5.001879024	5.731920793	1.912001953	3.041670168	3.188771958	2.858902312
MA16_Dca010993	WRKY12	7.449776341	8.735674087	4.637807941	2.211598508	1.94506934	0.815334818
MA16_Dca011569	WRKY44	1.557736703	1.567410433	0.951799973	0.508742472	0.912409837	0.631066031
MA16_Dca011860	WRKY08	5.928116257	6.390331648	0.504859418	2.603260905	2.782803735	1.399795922
MA16_Dca011912	WRKY37	1.069887793	1.098558151	0.639505891	0.675596224	0.715213347	0.215858427
MA16_Dca011914	WRKY38	0	0	0.158029011	0	0	0
MA16_Dca012410	WRKY60	75.65727335	75.50606841	49.34891724	50.29585481	51.86543932	51.78681789
MA16_Dca012846	WRKY51	0	0	0	1.023917641	2.725386186	0.420621327
MA16_Dca013149	WRKY61	3.003817113	2.594563989	3.849624046	11.89253274	16.51692303	13.82088816
MA16_Dca013510	WRKY64	82.53902201	82.24033941	70.4031078	83.82957568	85.78442625	79.17945686
MA16_Dca014563	WRKY18	1.016464499	1.474001951	0.698709057	0.341731714	1.041900316	0.786139423
MA16_Dca015482	WRKY56	54.85538294	52.73191034	29.07606366	55.63789159	51.05423582	52.8947148
MA16_Dca015639	WRKY15	2.999618173	4.474106068	8.522569748	4.1145203	3.382148063	2.7839061
MA16_Dca015848	WRKY55	3.983177924	4.340561099	2.100385594	2.971768199	3.187980504	1.645806653
MA16_Dca016437	WRKY22	8.264612142	9.305791764	3.475450061	2.941976037	4.33537594	3.15834961
MA16_Dca017113	WRKY48	3.844555664	2.606536268	0.427105436	2.256045047	1.49271254	1.306494471
MA16_Dca018137	WRKY03	32.11760866	33.19937346	29.47959375	43.93750277	49.96038307	43.53354253
MA16_Dca018897	WRKY35	4.25327919	5.126476647	2.953199963	7.331677955	6.407456181	6.778384877
MA16_Dca019319	WRKY40	3.263517283	2.433863518	1.345988425	0.394985805	0.37633308	0.662555991
MA16_Dca019656	WRKY32	1.726396532	1.457559324	0.492596729	0.078847922	0.400663598	0.302310542
MA16_Dca019840	WRKY53	1.969292472	1.060697077	0.72195995	0.953379443	0.50464292	0.456918598

MA16_Dca020108	WRKY19	66.5690276	70.06907281	27.26184772	37.77735151	40.19877192	34.9039383
MA16_Dca020159	WRKY42	0.436881325	0.543028389	0	0	0.403032386	0.202731901
MA16_Dca020342	WRKY43	0.698189686	0.3773155	1.085058352	1.726788	2.98710701	2.007331354
MA16_Dca020473	WRKY21	15.88750146	14.90000944	7.11130551	20.42123633	23.03895183	20.2886363
MA16_Dca021638	WRKY67	0	0	0	0.118570549	0	0
MA16_Dca022108	WRKY45	0.48342756	0	2.326127971	0.438821846	2.973148566	1.822692415
MA16_Dca023070	WRKY71	36.04850866	33.75938616	26.7985038	31.11794878	30.90945326	31.42045445
MA16_Dca024256	WRKY16	8.90849565	8.858361776	1.501275608	4.66061306	5.743957855	5.600806642
MA16_Dca024393	WRKY04	10.73005589	11.86799249	8.761689137	10.71469781	10.22652546	10.61980906
MA16_Dca027312	WRKY52	2.586337447	2.49141425	1.200032805	0.978207032	2.982439655	1.350194458
MA16_Dca028004	WRKY05	10.8226567	0.965798131	4.272887431	0	0.085334468	0.064386956
MA16_Dca028175	WRKY23	42.04831635	42.20594235	10.39860711	12.20605504	10.0665154	9.293791344
novel.1806	WRKY73	2.654751203	1.996495865	3.649706824	2.78105553	2.538246515	2.251618131
novel.2526	WRKY72	13.05972374	13.80582473	5.500700426	17.10409524	17.22531154	17.99145256
novel.574	WRKY74	0	0	2.821946631	0.124216766	0.47340312	1.309712437
