# Supplementary material for: Combined Analysis of Volatile Terpenoid Metabolism and Transcriptome Reveals Transcription Factors Related to Terpene Synthase in Two Cultivars of Dendrobium officinale Flowers
Source: Front Genet. 2021 Apr 22;12:661296. doi: 10.3389/fgene.2021.661296 (PMC8101708; doi:10.3389/fgene.2021.661296)
Supplement: Supplementary Table 6 — Gene IDs and relative transcript levels of DobHLH of two cultivals of D. officinale flower. [file Table_6.pdf]

Table S6 Gene IDs and relative transcript levels of *DobHLH* of two cultivals of *D. officinale* flower

Gene ID	Gene Name	Wanhu No.5-1	Wanhu No.5-2	Wanhu No.5-3	Wanhu No.6-1	Wanhu No.6-2	Wanhu No.6-3
MA16_Dca010117	bHLH01	229.9069252	247.8784961	100.7858239	167.618104	129.0970308	151.7361391
MA16_Dca017567	bHLH02	103.9973539	105.9808715	119.6294385	148.0292361	143.733151	165.7598544
MA16_Dca002144	bHLH03	102.7333313	107.8062559	108.9510548	107.7780455	113.5204634	123.795069
MA16_Dca006989	bHLH04	171.4940676	179.4477691	177.0532731	234.4486352	213.4101264	209.7994466
MA16_Dca011121	bHLH05	178.8998656	164.0886597	165.328346	219.7042532	186.3891877	263.620855
MA16_Dca013729	bHLH06	175.8271102	187.2170445	75.69321797	67.38022676	68.68036178	58.07616088
MA16_Dca011239	bHLH07	153.7137668	159.1345628	60.97150257	78.63305463	82.53807797	74.54681889
MA16_Dca016399	bHLH08	97.22936803	97.41827801	76.92837503	81.3283155	83.43398163	91.55524208
MA16_Dca000247	bHLH09	64.23054946	70.38127066	75.8190661	66.11528706	74.34169435	70.04685302
MA16_Dca022169	bHLH10	120.9888658	120.616597	62.57948849	41.44467554	46.04886828	30.54439908
MA16_Dca004599	bHLH11	57.60030153	65.88965536	65.00601793	59.14045569	56.46145302	53.25194428
MA16_Dca026004	bHLH12	34.11289908	35.4728614	34.1771795	47.35871287	54.33525119	45.09270126
MA16_Dca022885	bHLH13	60.29861019	59.51839387	35.70891267	19.89952591	17.61059606	19.15990231
MA16_Dca008361	bHLH14	41.15650024	41.93123562	12.67667504	17.5226651	15.04186957	13.46933119
MA16_Dca005153	bHLH15	24.25729768	23.06068311	23.45055779	21.68393832	20.77702429	21.9410615
MA16_Dca026267	bHLH16	70.75953989	67.09714472	22.0632812	31.86446702	35.48338462	12.07866268
MA16_Dca011185	bHLH17	24.21951592	20.65962244	18.68224329	21.66424614	22.91592052	20.51142868
MA16_Dca000511	bHLH18	7.914192587	8.165409282	9.991384242	15.99564139	18.49112586	16.02230757
MA16_Dca016470	bHLH19	24.00578404	23.84404655	13.94758816	12.3654016	14.77488522	9.57320196
MA16_Dca006524	bHLH20	20.90882442	19.63695335	13.8798975	10.5599217	9.582135439	7.338406293
MA16_Dca015651	bHLH21	8.246919071	8.721090324	9.111731184	10.53001493	10.25680416	9.993091984
MA16_Dca016133	bHLH22	18.09824785	15.65564967	7.228986689	12.72825415	11.9861641	7.34148287
MA16_Dca014460	bHLH23	4.773847156	4.919735705	11.17372186	14.11543605	16.90978247	15.56298908
MA16_Dca007145	bHLH24	4.511053686	4.399404238	5.311216548	6.103731893	10.70619363	7.138774118
MA16_Dca026460	bHLH25	8.880406772	8.620949946	3.564564165	10.70879277	8.770415695	9.136654229
MA16_Dca013905	bHLH26	10.07910917	12.85891226	6.431548366	8.792354972	13.68413489	6.795096293
MA16_Dca025545	bHLH27	14.66954244	16.3913012	1.264856711	5.876975055	17.68244855	17.37404375
MA16_Dca017853	bHLH28	9.841815063	10.09936693	5.821201413	15.9283269	19.44304318	18.52036646
MA16_Dca014975	bHLH29	11.83480305	13.05983276	7.698623153	10.13142998	6.035889779	16.67650893
MA16_Dca011715	bHLH30	5.414921376	5.579280111	6.268919458	5.432686989	4.43668709	5.248689813
MA16_Dca003414	bHLH31	4.843976489	6.450959326	14.27017828	5.758007949	2.925916273	3.813258225
MA16_Dca015800	bHLH32	3.602997091	4.027089151	1.919898896	1.554990012	4.122594772	1.846918265
MA16_Dca010986	bHLH33	3.43443184	2.613600052	5.781549195	3.774977815	1.832031586	3.211844691
MA16_Dca019447	bHLH34	6.377270417	6.209269274	1.656057448	1.629451714	1.688687293	2.17839593
MA16_Dca003888	bHLH35	6.182962959	6.128075372	2.333397121	2.200965822	2.050427263	1.547097817
MA16_Dca012252	bHLH36	2.53700676	3.416196035	3.923812986	2.644090943	2.004546181	2.493547138
MA16_Dca018524	bHLH37	1.14948331	1.964556039	17.97580004	14.43398821	11.0460728	14.50208863
MA16_Dca005413	bHLH38	1.58671009	2.070837096	2.835796676	3.456731598	2.622595198	3.405398429
MA16_Dca001510	bHLH39	2.942322095	1.760876327	1.747863012	2.63786166	2.513291844	2.233467737
MA16_Dca017063	bHLH40	0	0	18.55801219	1.922091011	11.33675892	10.00144043
MA16_Dca007274	bHLH41	1.966955663	1.768620618	2.531532706	2.684529331	1.672997433	2.913040902
MA16_Dca003689	bHLH42	0.78373862	0.865920017	1.616205798	3.846955601	2.67783583	3.63688743
MA16_Dca024215	bHLH43	0.537098831	0.770302252	0.965592601	2.85023902	2.223330946	1.773418351
MA16_Dca011113	bHLH44	1.542311321	3.195066119	0.271838896	0.813676797	1.520101761	0.367025337
MA16_Dca003699	bHLH45	2.132594035	1.917557091	0.810938348	1.153254606	0.767411373	1.368618164

MA16_Dca015147	bHLH46	0.30525442	1.276233244	1.411578883	0.940422211	0.554674042	0.740449989
MA16_Dca009217	bHLH47	0.919003154	1.14228916	1.173184919	2.224552033	1.413000175	1.751521294
MA16_Dca015677	bHLH48	0.235121586	0.657558013	0.888913189	0.569138637	0.677827194	0.716012213
MA16_Dca025889	bHLH49	0.643012072	0.888046427	1.571559229	2.161783497	1.42805582	0.497309193
MA16_Dca017158	bHLH50	1.530377187	1.521764764	1.577952406	0.83350185	0.470601918	0.266310544
MA16_Dca023074	bHLH51	0.948070912	0.565641302	1.042713418	0.367186217	0	0.087989212
MA16_Dca013486	bHLH52	0.687855704	0.683984694	0.819565351	0.222004433	0.493547933	0.531991512
MA16_Dca021299	bHLH53	0.34950506	0.530961092	0.320329077	0.658013139	0.298542508	0.630721469
MA16_Dca014641	bHLH54	0.676166653	0.630338836	0.232395605	0.460332721	0.064976899	0.39221335
MA16_Dca023381	bHLH55	0.855403436	0.562890128	0.415056742	0.426300341	0.232096471	0
MA16_Dca026923	bHLH56	0.295822911	0.315169418	0.755285716	0	0.324884494	0.343186681
MA16_Dca009652	bHLH57	0.214633813	0.533564824	0.073768729	0.194829616	0.825017885	0.435747405
MA16_Dca015531	bHLH58	0.1328598	0.16514014	0.669729115	0.321602312	0.136184459	0.4110181
MA16_Dca018902	bHLH59	0.124943838	0.155300873	0.515311993	0.453661232	0.192105614	0.869690472
MA16_Dca029163	bHLH60	0.67177596	0.626245727	0.230886543	2.235901788	1.291099418	0
MA16_Dca004286	bHLH61	0.284212906	0.52990023	0.195365536	1.719924452	0.655481243	0.164858908
MA16_Dca000058	bHLH62	0.048983664	0.182655004	0.269367633	0.237141099	0.301256531	0.056826366
MA16_Dca011622	bHLH63	0.5829859	0.2635023	0.072861737	0	0	0
MA16_Dca026399	bHLH64	0.078137083	0.048560847	0	0.023642466	0.120138556	0.090647496
MA16_Dca005213	bHLH65	0	0	0	0.240789423	0	0.519305561
MA16_Dca011038	bHLH66	0	0	0.419958987	0	0.156558512	0.295318123
MA16_Dca009148	bHLH67	0	0.096249343	0	0.140580651	0.059529734	0.179666595
MA16_Dca006152	bHLH68	0.351086531	0.072731404	0	0	0.179936027	0
MA16_Dca002562	bHLH69	0.158347191	0.328033476	0	0	0	0
MA16_Dca021380	bHLH70	0.119369421	0	0.16410705	0.096315769	0	0
MA16_Dca002730	bHLH71	0	0	0.846583989	0	0	0
MA16_Dca020095	bHLH72	0	0	0.097148982	0.042763149	0	0.12296853
MA16_Dca027196	bHLH73	0.084520832	0	0	0	0	0
MA16_Dca000594	bHLH74	0	0	0	0.064408693	0.081822761	0
MA16_Dca017997	bHLH75	0	0	0	0.090470015	0	0.086717692
MA16_Dca024791	bHLH76	0	0	0.18328107	0	0	0
MA16_Dca020549	bHLH77	0	0	0	0.042415481	0	0
MA16_Dca027218	bHLH78	0	0	0	0.053055297	0	0
MA16_Dca020024	bHLH79	0	0	0	0	0	0
MA16_Dca012686	bHLH80	0	0	0	0	0	0
MA16_Dca009639	bHLH81	0	0	0	0	0	0
MA16_Dca021694	bHLH82	0	0	0	0	0	0
MA16_Dca006424	bHLH83	0	0	0	0	0	0
MA16_Dca023137	bHLH84	0	0	0	0	0	0
MA16_Dca009809	bHLH85	0	0	0	0	0	0
MA16_Dca014221	bHLH86	0	0	0	0	0	0
